# Renal Mitochondrial ATP Transporter Ablation Ameliorates Obesity-Induced CKD

**DOI:** 10.1681/ASN.0000000000000294

**Published:** 2024-01-11

**Authors:** Anna Permyakova, Sharleen Hamad, Liad Hinden, Saja Baraghithy, Aviram Kogot-Levin, Omri Yosef, Ori Shalev, Manish Kumar Tripathi, Haitham Amal, Abhishek Basu, Muhammad Arif, Resat Cinar, George Kunos, Michael Berger, Gil Leibowitz, Joseph Tam

**Affiliations:** 1 Obesity and Metabolism Laboratory, Faculty of Medicine, School of Pharmacy, The Institute for Drug Research, The Hebrew University of Jerusalem, Jerusalem, Israel; 2 Diabetes Unit and Endocrine Service, Hadassah-Hebrew University Medical Center, Jerusalem, Israel; 3 The Lautenberg Center for Immunology and Cancer Research, Faculty of Medicine, Israel-Canada Medical Research Institute, The Hebrew University of Jerusalem, Jerusalem, Israel; 4 Metabolomics Center, Core Research Facility, Faculty of Medicine, The Hebrew University of Jerusalem, Jerusalem, Israel; 5 The Laboratory of Neuromics, Cell Signaling and Translational Medicine, Faculty of Medicine, School of Pharmacy, Institute for Drug Research, The Hebrew University of Jerusalem, Jerusalem, Israel; 6 Section on Fibrotic Disorders, National Institute on Alcohol Abuse and Alcoholism, National Institutes of Health, Rockville, Maryland; 7 Laboratory of Cardiovascular Physiology and Tissue Injury, National Institute on Alcohol Abuse and Alcoholism, National Institutes of Health, Rockville, Maryland; 8 Laboratory of Physiologic Studies, National Institute on Alcohol Abuse and Alcoholism, National Institutes of Health, Rockville, Maryland

**Keywords:** CKD, metabolism, mitochondria, obesity

## Abstract

**Significance Statement:**

This study sheds light on the central role of adenine nucleotide translocase 2 (ANT2) in the pathogenesis of obesity-induced CKD. Our data demonstrate that ANT2 depletion in renal proximal tubule cells (RPTCs) leads to a shift in their primary metabolic program from fatty acid oxidation to aerobic glycolysis, resulting in mitochondrial protection, cellular survival, and preservation of renal function. These findings provide new insights into the underlying mechanisms of obesity-induced CKD and have the potential to be translated toward the development of targeted therapeutic strategies for this debilitating condition.

**Background:**

The impairment in ATP production and transport in RPTCs has been linked to the pathogenesis of obesity-induced CKD. This condition is characterized by kidney dysfunction, inflammation, lipotoxicity, and fibrosis. In this study, we investigated the role of ANT2, which serves as the primary regulator of cellular ATP content in RPTCs, in the development of obesity-induced CKD.

**Methods:**

We generated RPTC-specific ANT2 knockout (*RPTC-ANT2*^−/−^) mice, which were then subjected to a 24-week high-fat diet–feeding regimen. We conducted comprehensive assessment of renal morphology, function, and metabolic alterations of these mice. In addition, we used large-scale transcriptomics, proteomics, and metabolomics analyses to gain insights into the role of ANT2 in regulating mitochondrial function, RPTC physiology, and overall renal health.

**Results:**

Our findings revealed that obese *RPTC-ANT2*^−/−^ mice displayed preserved renal morphology and function, along with a notable absence of kidney lipotoxicity and fibrosis. The depletion of Ant2 in RPTCs led to a fundamental rewiring of their primary metabolic program. Specifically, these cells shifted from oxidizing fatty acids as their primary energy source to favoring aerobic glycolysis, a phenomenon mediated by the testis-selective Ant4.

**Conclusions:**

We propose a significant role for RPTC-Ant2 in the development of obesity-induced CKD. The nullification of RPTC-Ant2 triggers a cascade of cellular mechanisms, including mitochondrial protection, enhanced RPTC survival, and ultimately the preservation of kidney function. These findings shed new light on the complex metabolic pathways contributing to CKD development and suggest potential therapeutic targets for this condition.

## Visual Abstract

**Figure:**
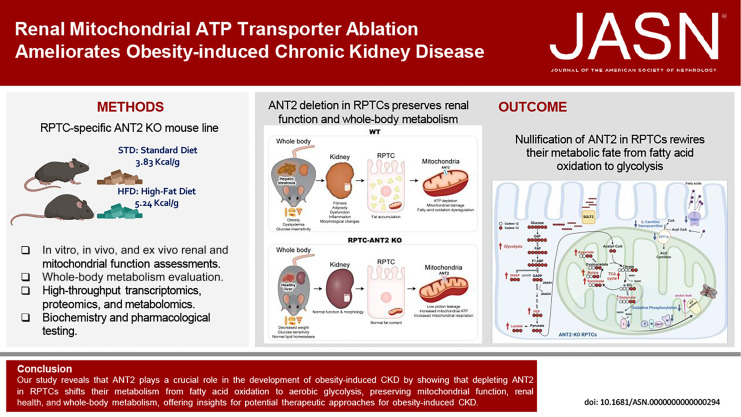

## Introduction

CKD is a global public health concern because of its rapidly growing prevalence and associated mortality. CKD incidence has increased in the past two decades, leading to adverse outcomes, such as ESKD and higher mortality rate.^1^ Moreover, progressive loss of kidney function in patients with CKD is linked with metabolic complications, such as obesity, diabetes, cardiovascular disease, inflammation, and fatty liver.^2–5^ Obesity, which has also reached epidemic proportions,^6^ is a primary contributor to CKD and has been associated with it for almost a century.^7^ The renal proximal tubule cells (RPTCs) are a primary site of kidney injury in obesity,^8–10^ but the mechanisms of RPTC injury are not fully understood, highlighting a critical need to elucidate the etiology of obesity-induced kidney dysfunction to facilitate the development of novel therapeutics.

The kidney is a highly energy-demanding organ, with the second highest mitochondrial content and oxygen consumption after the heart.^11^ Mitochondria generate ATP through oxidative phosphorylation (OXPHOS) in the kidney, providing energy for cellular functions. Although all kidney structures have a great need for ATP, the metabolic routes underlying ATP production are cell-type specific. RPTCs, which constitute approximately 90% of the outer kidney cortex and are responsible for up to 80% of nutrient reabsorption, serve as the primary ATP-consuming cells in the kidney. These cells use fatty acids through mitochondrial *β*-oxidation.^12–14^ RPTCs also have the highest mitochondria density in the kidney.^15^ CKD leads to mitochondrial dysfunction, which further impairs RPTC function and glucose and lipid metabolism and can initiate kidney injury, inflammation, and fibrosis by oxidative stress and changes in ATP levels.^16–18^ Impairments in mitochondrial biogenesis, architecture, metabolism, and respiration can act as an initiating insult for RPTC dysfunction.

Adenine nucleotide translocators (ANTs) are crucial for regulating ATP levels by exchanging cytosolic and mitochondrial adenine nucleotides across the inner mitochondrial membrane, providing ADP for ATP synthesis and delivering ATP into the cytosol.^19,20^ Four ANT isoforms are present in humans,^21^ whereas only three exist in mice, with each isoform having a specific expression pattern depending on tissue, cell type, status of proliferation, and developmental stage.^21,22^ In mouse RPTCs, ANT1 (*Slc25a4*) and ANT2 (*Slc25a5*) are the predominant isoforms while ANT4 (*Slc25a31*) is barely detected.^23^ Although the role of ANT2 in animal development has not yet been demonstrated,^24^ several tissue-specific ANT2 knockout mice have been developed and characterized.^25–27^ Liver-specific ANT2 knockout mice are leaner; resistant to hepatic steatosis, obesity, and insulin resistance^25^; and its selective nullification in white adipose tissue reduces adipocyte hypoxia and improves insulin sensitivity.^26,27^ In addition, blocking ANT2 in the liver markedly reduces fatty acid–stimulated OXPHOS.^28^ However, the role of ANT2 in the kidney and its contribution to obesity-induced CKD development have not yet been reported. In this study, we show that nullification of ANT2, specifically in RPTCs in mice, results in full protection from obesity-induced renal dysfunction, adiposity, and fibrosis as well as improves whole-body metabolism. Thus, our findings indicate that targeting ANT2 in RPTCs has the potential to treat obesity-induced CKD.

## Methods

Methodologies, including animals and cell culture experiments, metabolic and skeletal assessments, microscopy, biochemistry, flow cytometry, mitochondrial function assessment, transcriptomics, proteomics, metabolomics, and statistical analysis, are detailed in the Supplemental Methods and Supplemental Excel Sheet 2.

## Results

### Lipotoxic Conditions Reduce Renal ANT2 Expression in RPTCs

To decipher the role of ANT2 in the kidney, we first assessed its localized expression in lineage-traced RPTCs (YFP+ cells) by using our Sglt2-Cre; Rosa26-YFP reporter mice.^29^ As depicted in Supplemental Figure 1, A and B, ANT2 is expressed in RPTCs and other renal cells, and a trend toward its reduced expression was found when these mice were fed with a high-fat diet (HFD) for 24 weeks. Next, we measured ANT2 expression levels in three models of lipotoxicity: (*1*) kidney lysates collected from HFD-fed WT mice for 24 weeks, (*2*) primary mouse RPTCs, and (*3*) HK-2 cell line. Both cell cultures were exposed to fatty acid flux conditions: 0.1 and 0.25 mM, for 48 and 24 hours oleate:palmitate (O:P, 2:1), respectively. In all these experimental settings, ANT2 expression was significantly reduced relative to the respective controls (Supplemental Figure 1, C–H), suggesting a cellular mechanism linking mitochondrial dysfunction and obesity-induced CKD.

### Generating RPTC-Specific ANT2-Null Mice

To assess this hypothesis and to specifically explore the role of ANT2 in modulating kidney and mitochondrial function in obesity, we generated RPTC-specific ANT2-null mice (*RPTC-ANT2*^−/−^) by crossing ANT2-floxed mice^30^ with transgenic iL1-Sglt2-Cre mice,^31^ in which *Cre* recombinase is expressed in the nucleus of the *S1* segment of the proximal tubule (Supplemental Figure 1I). Gene and protein expression analysis revealed that ANT2 deletion occurred specifically in RPTCs isolated from mouse kidneys (Supplemental Figure 1J), whereas ANT2 expression remained unchanged in the brain, liver, and pancreas (Supplemental Figure 1K).

### *RPTC-ANT2*^−/−^ Mice are Protected from Obesity-Induced Kidney Injury and Interstitial Fibrosis

**Figure 1:**
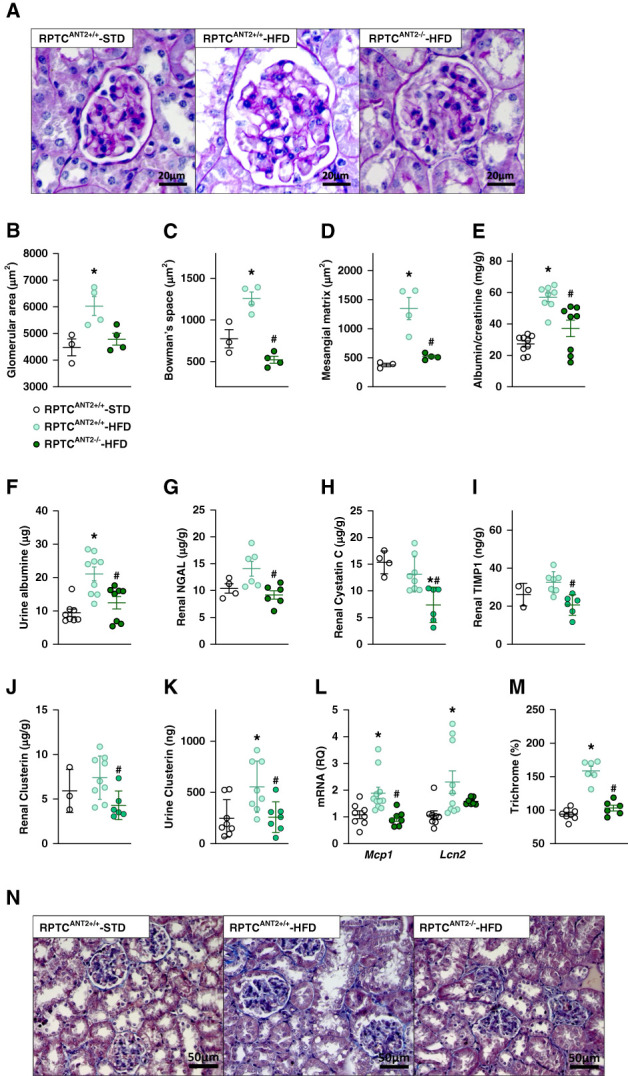
***RPTC-ANT2***^**−/−**^ **mice are protected from obesity-induced kidney injury and interstitial fibrosis.** *RPTC-ANT2*^−/−^ mice and their WT littermate controls were fed either a STD or a HFD for 24 weeks. (A–D) Kidney PAS staining (A) and the quantification of glomerular (B), Bowman's space (C), and mesangial matrix (D) areas (*n*=3–4 mice per group). (E and F) Biochemical measurements of urinary albumin-to-creatinine ratio (E) and the urine albumin levels (F) (*n*=8–9 mice per group). (G–K) Assessment of the kidney injury markers: renal NGAL (G), cystatin C (H), TIMP1 (I), clusterin (J), and urine clusterin (K) (*n*=3–9 mice per group). (L) Gene expression levels of renal *Mcp1* and *Lcn2* (*n*=7–10 mice per group). (M and N) Trichrome renal fibrogenesis quantification (M) and its representative staining (N) (*n*=6–8 mice per group). The data represent mean±SEM. *P < 0.05 versus RPTC^ANT2+/+^-STD, #*P* < 0.05 versus RPTC^ANT2+/+^-HFD by one-way ANOVA. ANT2, adenine nucleotide transporter 2; HFD, high-fat diet; NGAL, neutrophil gelatinase-associated lipocalin; PAS, periodic acid–Schiff; RPTC, renal proximal tubule cell; STD, standard diet; TIMP1, tissue inhibitor of metalloproteinase 1; WT, wild type.

Once generated, male *RPTC-ANT2*^−/−^ mice and their wild type (WT) littermate controls were fed with a HFD for 24 weeks. Then, we compared the renal phenotype of both strains with WT mice fed with a standard diet (STD) to assess the effect of ANT2 deficiency on the development of obesity-induced CKD. Comparing their kidney morphology and function, we found that the HFD-fed WT but not *RPTC-ANT2*^−/−^ mice had larger glomerular and Bowman's space areas (Figure 1, A–C), increased mesangial expansion (Figure 1, A and D), as well as elevated albumin-to-creatinine ratio and urinary albumin levels (Figure 1, E and F). In addition, renal neutrophil gelatinase-associated lipocalin (NGAL) (Figure 1G), cystatin C (Figure 1H), tissue inhibitor of metalloproteinase (TIMP) metallopeptidase inhibitor 1 (TIMP1; Figure 1I), clusterin (Figure 1J), and urinary clusterin (Figure 1K) levels, all of which are known markers for kidney/RPTC injury, were reduced in HFD-fed *RPTC-ANT2*^−/−^ mice in comparison with their obese WT littermates. The HFD-induced upregulation in the expression levels of the inflammatory markers monocyte chemotactic protein-1 (*Mcp1*) and lipocalin 2 (*Lcn2*) in WT controls was reduced in the obese null mice (Figure 1L). Furthermore, renal fibrosis was significantly reduced in obese *RPTC-ANT2*^−/−^ mice compared with WT HFD-fed mice (Figure 1, M and N). Taken together, these findings suggest a key role of RPTC-ANT2 in mediating obesity-induced renal dysfunction, inflammation, and tubulointerstitial fibrosis.

### RPTC-ANT2 Nullification Alters Cellular and Organelle Functions at Both the Transcriptome and Proteome Levels

**Figure 2:**
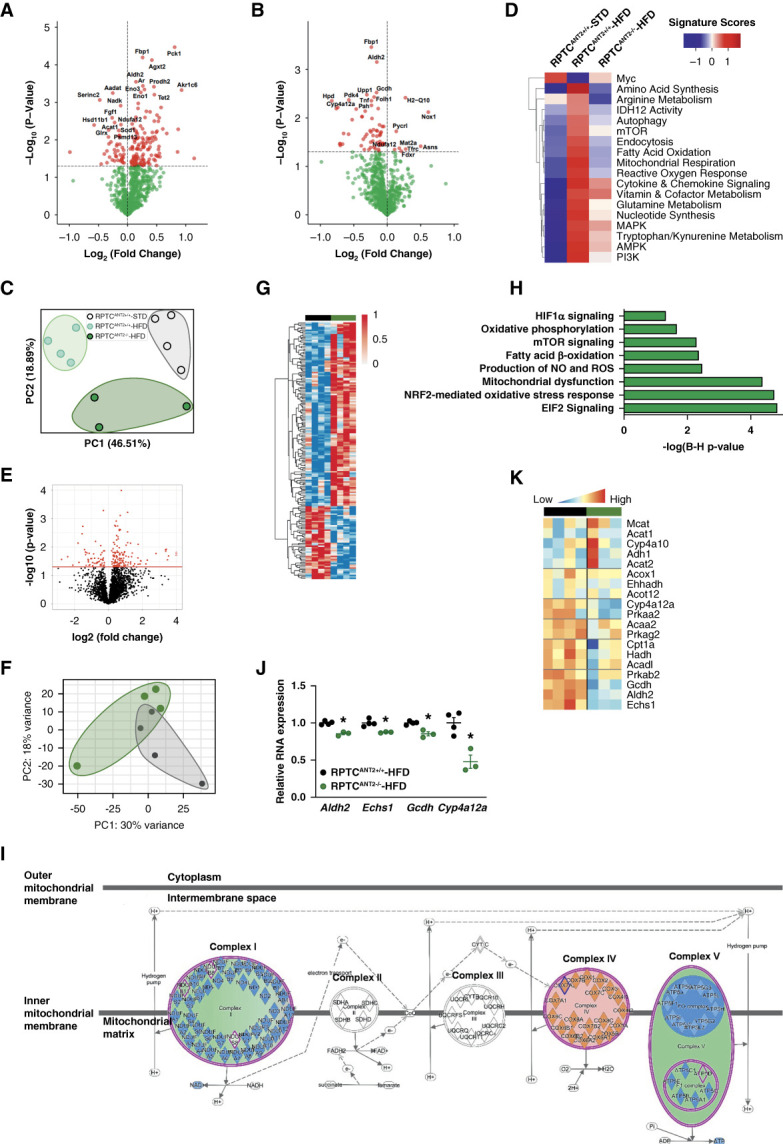
**RPTC-ANT2 nullification alters cellular and organelle functions at both the transcriptome and proteome levels.** *RPTC-ANT2*^−/−^ mice and their WT littermate controls were fed either a STD or a HFD for 24 weeks. (A) A volcano plot of differentially expressed genes in the kidney of RPTC-ANT2^+/+^-HFD versus RPTC-ANT2^+/+^-STD mice. (B) A volcano plot of differentially expressed genes in the kidney of *RPTC-ANT2*^−/−^-HFD versus RPTC-ANT2^+/+^-HFD mice. (C) A PCA of the global transcriptomic profile of the three treatment groups. (D) A heatmap characterization of the signaling pathways affected in the three treatment groups. The data were collected from 3 to 4 animals per group. (E) A volcano plot of differentially expressed proteins (*P* value < 0.05) that participate in Qiagen IPA in the kidney of *RPTC-ANT2*^−/−^-HFD versus RPTC-ANT2^+/+^-HFD mice. (F) A PCA of the global proteomics response to ANT2 deletion in RPTCs under HFD conditions. The first two PCs are shown. (G) A heatmap of the 176 differentially expressed proteins between *RPTC-ANT2*^−/−^-HFD and RPTC-ANT2^+/+^-HFD (*P* value < 0.05). The heatmap was drawn after scaling per protein (rows) the log2 LFQ intensity values over all the samples. Proteins are ordered by hierarchical clustering. (H) A log (B–H *P* value) of selected significantly enriched Qiagen IPA canonical pathways (B–H *P* value < 0.05) in the kidney of *RPTC-ANT2*^−/−^-HFD versus RPTC-ANT2^+/+^-HFD mice. (I) The Qiagen IPA OXPHOS canonical pathway. Differentially expressed proteins (*P* value < 0.05) of the *RPTC-ANT2*^−/−^-HFD versus RPTC-ANT2^+/+^-HFD comparison (in their gene symbols) that participate in this pathway (purple outlines) are colored in pink (upregulated) and green (downregulated), whereas the predicted proteins (by the Qiagen IPA molecular activity predictor tool) are depicted in orange and blue (upregulated and downregulated, respectively). (J) The mRNA expression levels of selected representative genes related to the OXPHOS pathway. Data are from 3 to 4 mice per group. The data represent mean±SEM. *P < 0.05 versus RPTC-ANT2^+/+^-HFD by Student *t* test. (K) A heatmap of the differentially expressed genes in the OXPHOS pathway between *RPTC-ANT2*^−/−^-HFD and ^+/+^-HFD mice. AMPK, AMP-activated protein kinase; IPA, ingenuity pathway analysis; LFQ, label-free quantification; OXPHOS, oxidative phosphorylation; PC, principle component; PCA, principal component analysis; ROS, reactive oxygen species.

To understand how the nullification of ANT2 results in renal protection under HFD conditions, we facilitated several omics analyses. Using targeted large-scale transcriptomics (Supplemental Source Data A), we found that HFD-fed WT mice had 45 downregulated genes and 132 upregulated genes in the kidney compared with STD-fed WT animals (Figure 2A). In addition, 61 genes were downregulated and eight genes were upregulated in HFD-fed *RPTC-ANT2*^−/−^ animals, compared with their HFD-fed WT controls (Figure 2B). Principal component analysis (Figure 2C) and heatmap characterization of the signaling pathways associated with these genes (Figure 2D) showed clear differences in the transcriptomic profiles of the three groups of animals, with ANT2 deletion in RPTCs ameliorating the HFD-induced upregulation in several signaling pathways, including amino acid synthesis, mitochondrial respiration, reactive oxygen response, endocytosis, fatty acid oxidation (FAO), mammalian target of rapamycin, AMP-activated protein kinase (AMPK) signaling, and others (Supplemental Figure 2), suggesting altered cellular and organelle functions in the absence of ANT2 in RPTCs.

We next mapped the entire proteome of renal cortices from HFD-fed *RPTC-ANT2*^−/−^ mice and their HFD- and STD-fed WT littermates to investigate the biological processes and cellular pathways in obesity-induced CKD affected by ANT2 deletion. By applying mass spectrometry, followed by a large-scale system biology analysis together with bioinformatics, we identified 176 proteins that were either significantly upregulated (126) or downregulated (50) (Figure 2E and Supplemental Methods) in the absence of ANT2. Focusing on the differentially expressed proteins, unsupervised principal component analysis (Figure 2F) and hierarchical clustering analysis (Figure 2G) showed a separation between the HFD-fed WT and HFD-fed *RPTC-ANT2*^−/−^ mice, indicating that the proteomic profiles contain a structure that is discernible even without considering the identity of the individual proteins. By using bioinformatics, followed by enrichment of canonical pathways, we found that most of these modulated proteins are associated with several biological processes (*e.g*., reactive oxidative stress response, mitochondrial dysfunction, FAO, mitochondrial OXPHOS/respiration, and mammalian target of rapamycin complex signaling; Figure 2H), findings that were directly linked with the transcriptomic analysis (Figure 2D).

Specifically, six of ten proteins (*ACO2*, *ATP5F1D*, *MAOB*, *NDUFB11*, *NDUFB8*, *NDUFS8*) were found to be downregulated in obese *RPTC-ANT2*^−/−^ mice, contributing to the significant reduction in the mitochondrial dysfunction pathway. Furthermore, the FAO pathway was also significantly altered. We identified four affected proteins associated with this pathway; two proteins (*ACAA2*, *ECI1*), associated with the mitochondrial FAO, were downregulated, whereas the other two proteins (*EHHADH*, *HSD17B4*), associated with peroxisomal FAO, were significantly upregulated, suggesting that mitochondrial and peroxisomal metabolic activities are closely intertwined. Furthermore, we found that one of the most significantly altered pathways after ANT2 nullification is the mitochondrial OXPHOS pathway (Figure 2I), which was robustly downregulated. Regarding this pathway, four proteins that belong to complex I (*NDUFB11*, *NDUFB8*, *NDUFS8*) and complex V (*ATP5F1D*) were downregulated while only one protein (*COX7A2L*) that belongs to complex IV was upregulated. In fact, our results from the large-scale RNA transcriptomic analysis, which also demonstrated the downregulated expression of the OXPHOS-related genes, further support this observation (Figure 2, J and K and Supplemental Figure 2). Taken together, deletion of ANT2 in RPTCs affects the transcription of genes and their protein translation, which may account for the changes in mitochondrial function and respiration.

### ANT2 Nullification Reduces Renal Lipotoxicity by Inhibiting Fatty Acid Transport and their Cellular Utilization in RPTCs

**Figure 3:**
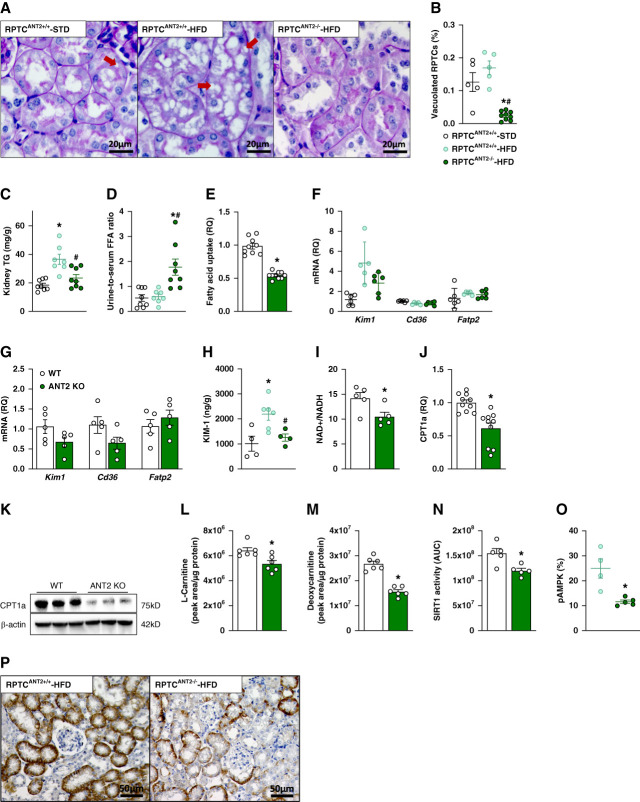
**Decreased fatty acid transport, utilization, and accumulation in ANT2-deleted RPTCs.** *RPTC-ANT2*^−/−^ mice and their WT littermate controls were fed a HFD for 24 weeks. (A and B) PAS staining (A) and quantification (B) of fat vacuolated RPTCs (*n*=5–9 mice per group). Red arrows denote the fat vacuoles. (C) Kidney TG levels (*n*=7–8 mice per group). (D) Urine-to-serum FFA ratio (*n*=7–8 mice per group). (E) Fatty acid uptake by primary mouse RPTCs isolated from STD-fed *RPTC-ANT2*^−/−^ and WT mice (*n*=9–10 biological replicates in each group). (F and G) The gene expression levels of the fatty acid transporters: *Kim1*, *Cd36*, and *Fatp2* in kidney cortices (F) and primary mouse RPTCs (G) (*n*=5–6 biological replicates in each group). (H) Protein levels of KIM-1 in kidney cortices collected from mice (*n*=4–6 mice per group). (I) The NAD^+^/NADH ratio measured in primary mouse RPTCs isolated from STD-fed *RPTC-ANT2*^−/−^ and WT mice (*n*=5 biological replicates per group). (J and K) CPT1a protein expression levels in primary mouse RPTCs isolated from STD-fed *RPTC-ANT2*^−/−^ and WT mice quantified (J) by Western blotting (K) (*n*=10 biological replicates in each group). (L and M) l-carnitine (L) and deoxycarnitine (M) metabolite levels in primary mouse RPTCs isolated from STD-fed *RPTC-ANT2*^−/−^ and WT mice, exposed for 3 hours to O:P (2:1, 0.1 mM), and measured using LC-MS-based metabolomics analysis (*n*=6 biological replicates in each group). (N) SIRT1 activity in primary mouse RPTCs isolated from STD-fed *RPTC-ANT2*^−/−^ and WT mice (*n*=5 biological replicates per group). (O and P) Quantification of kidney phosphorylated AMPK (O) by immunohistochemical staining (P) in *RPTC-ANT2*^−/−^ mice and their WT controls fed with a HFD (*n*=4–5 mice per group). The data represent mean±SEM. *In vivo*: *P < 0.05 versus RPTC^ANT2+/+^-STD, #*P* < 0.05 versus RPTC^ANT2+/+^-HFD by one-way ANOVA. *In vitro*: *P < 0.05 versus WT by Student *t* test. FFA, free fatty acid; KIM-1, Kidney injury marker 1; KO, knockout; LC-MS, liquid chromatography–mass spectrometry; NADH, nicotinamide adenine dinucleotide hydrogen; O:P, oleate:palmitate; SIRT1, sirtuin1; TG, triglyceride.

Triggered by the reduced FAO in the absence of ANT2, found in both the transcriptomics and proteomics analyses, we next analyzed the lipid profile of the mice and primary RPTCs. Lipid accumulation in the kidney, and specifically in RPTCs, is a main feature of obesity-induced CKD,^16,17,32,33^ and quantifying vacuolated proximal tubules revealed a high accumulation of lipid droplets in HFD-fed WT mice, but not in the obese *RPTC-ANT2*^−/−^ mice (Figure 3, A and B). The beneficial effect of ANT2 deletion in RPTCs was accompanied by reduced total kidney triglyceride accumulation (Figure 3C) as well as increased urine-to-serum free fatty acid ratio (Figure 3D) in the HFD-fed *RPTC-ANT2*^−/−^ mice, suggesting enhanced urinary lipid excretion that reduces fat accumulation in RPTCs. Verifying this effect *in vitro*, we found a significant reduction of fatty acid uptake in primary mouse RPTCs lacking ANT2 (Figure 3E). To better understand the mechanism underlying this phenomenon, we measured, both *in vivo* and *in vitro*, the expression of genes responsible for lipid transport in RPTCs, such as kidney injury marker-1 (*Kim1*), cluster of differentiation 36 (*Cd36*), and fatty acid transport protein 2 (*Fatp2*). While none of them showed significant changes (Figure 3, F and G), only *Kim1* displayed a tendency toward reduced expression in the absence of ANT2. Indeed, verifying this change at the protein level, we found that the HFD-induced upregulation of KIM-1 in WT animals was normalized in obese RPTC-ANT2 null mice (Figure 3H), suggesting KIM-1 in mediating the reduced fatty acid uptake in ANT2-deleted RPTCs.

**Figure 4:**
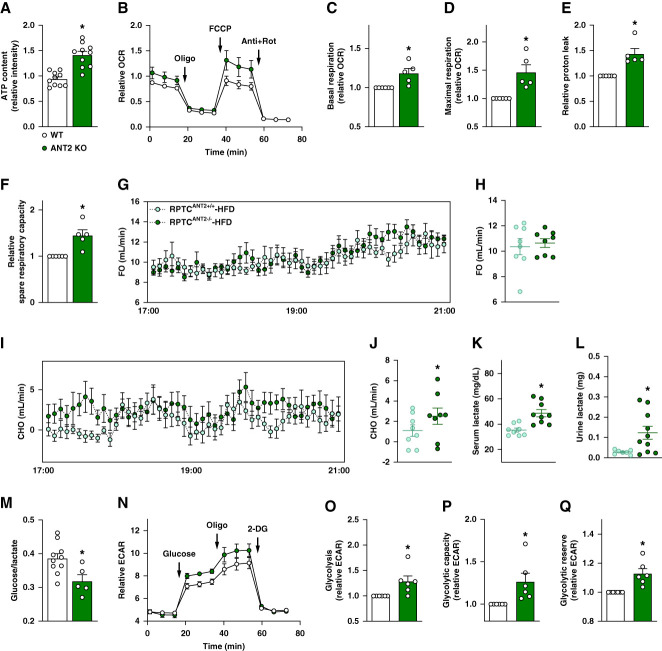
**Nullification of ANT2 increases aerobic glycolysis in RPTCs.** (A) Cellular ATP content measured in primary mouse RPTCs isolated from STD-fed *RPTC-ANT2*^−/−^ and WT mice (*n*=10 biological replicates per group). (B–F) Seahorse OCR measurement (B) in primary mouse RPTCs isolated from STD-fed *RPTC-ANT2*^−/−^ and WT mice, from which basal respiration (C), maximal respiration (D), proton leak (E), and spare respiratory capacity (F) were calculated (*n*=5–6 biological replicates per group). (G–J) Metabolic measurements of FO (G and H) and CHO (I and J) measured over a period of 4 hours shown over time (G and I) and in total (H and J) in *RPTC-ANT2*^−/−^ mice and their WT controls fed with a HFD (*n*=8 mice per group). (K and L) Biochemical lactate measurement in serum (K) and urine (L) in *RPTC-ANT2*^−/−^ mice and their WT controls fed with a HFD (*n*=7–10 mice per group). (M) Biochemical measurements of the glucose-to-lactate ratio in primary mouse RPTCs isolated from STD-fed *RPTC-ANT2*^−/−^ and WT mice (*n*=5–10 biological replicates per group). (N–Q) The Seahorse ECAR measurement (N) in primary mouse RPTCs isolated from STD-fed *RPTC-ANT2*^−/−^ and WT mice, from which glycolysis (O), glycolytic capacity (P), and glycolytic reserve (Q) were calculated (*n*=6 biological replicates per group). The data represent mean±SEM. *In vitro*: *P < 0.05 versus WT by Student *t* test. *In vivo*: *P < 0.05 versus RPTC^ANT2+/+^-HFD by Student *t* test. CHO, carbohydrate oxidation; ECAR, extracellular acidification rate; FO, fat oxidation; OCR, oxygen consumption rate.

In addition, the transcriptomics and proteomics analyses further suggested cellular and mitochondrial changes that could modulate cellular fatty acid utilization unrelated to their transport into the RPTCs (Figure 2). Interestingly, we found reduced ratio between NAD^+^ and nicotinamide adenine dinucleotide hydrogen in ANT2-deleted RPTCs (Figure 3I), which could also point toward inhibition of OXPHOS. Moreover, a significant downregulation in the expression of carnitine palmitoyl transferase I (CPT1; Figure 3, J and K), which catalyzes the transport of long-chain fatty acids into the mitochondria for *β*-oxidation, was found in ANT2-deleted RPTCs. Large-scale metabolomics analysis, performed on primary mouse WT and ANT2 knockout (KO) RPTCs, extracted from STD-fed animals and exposed to O:P (2:1, 0.1 mM) for 3 hours, revealed a significant reduction in the levels of l-carnitine (Figure 3L) and deoxycarnitine (Figure 3M) in the absence of ANT2, both required for the proper function of CPT1a. These findings were further coupled with significant reductions in the activity of sirtuin1 (SIRT1) (Figure 3N) and phosphorylated AMPK (Figure 4, O and P), both of which are regulators of fatty acid *β*-oxidation–related OXPHOS, suggesting (and further supporting) the reduction in OXPHOS in the absence of ANT2. Altogether, these findings suggest an impairment in the cellular/mitochondrial utilization of fatty acids in RPTCs lacking ANT2.

### Altered Mitochondrial Functions in the Absence of RPTC-ANT2

Measurements of cellular ATP revealed an unexpected significant elevation in its content in primary RPTCs collected from STD-fed ANT2^−/−^ and WT mice (Figure 4A). This elevation was coupled with increased basal and maximal oxygen consumption rates (OCRs; Figure 4, B–D), increased proton leakage (Figure 4E), and enhanced spare respiratory capacity (Figure 4F). Dichlorodihydrofluorescein diacetate staining for reactive oxygen species production did not reveal any changes between the genotypes (Supplemental Figure 3A), and no significant changes in nitric oxide metabolites, nitrite, and nitrate were found in primary mouse RPTCs collected from STD-fed WT and ANT2-null mice and their media (Supplemental Figure 3, B–E). Furthermore, because nitric oxide plays a major role in pathology-induced oxidative/nitrosative stress,^34^ we also assessed 3-nitrotyrosine expression and found no difference in its production between the null and WT groups (Supplemental Figure 3, F and G). Taken together, these findings suggest that nullification of ANT2 in RPTCs does not induce any changes in cellular oxidative/nitrosative stress.

To decipher whether the increased ATP levels and mitochondrial respiration were mediated by changes in mitochondrial biomass, we used a flow cytometric analysis to track mitochondrial mass in *RPTC-ANT2*^−/−^ animals using a novel mouse model in which the photo-convertible fluorescent protein mito-Dendra2^35^ was used. Our findings show that mitochondrial biomass was unaffected by the nullification of ANT2 (Supplemental Figure 4, A and B). The RPTCs, extracted from STD-fed animals, were also subjected to tetramethylrhodamine staining to assess their mitochondrial membrane potential (Supplemental Figure 4, C and D); however, no difference between WT and ANT2 KO cells was noted. In addition, transmission electron microscopy analysis revealed no variation in mitochondrial morphology (Supplemental Figure 4, E–G) in RPTCs, and no changes were measured in the expression levels of key mediators related to mitochondrial fission and fusion (Supplemental Figure 4, H–J). Taken together, these data suggest that the increased production of ATP in RPTCs lacking ANT2 is not associated with any changes in mitochondrial biogenesis, mass, and architecture.

### Nullification of ANT2 in RPTCs Increases Cellular Glycolysis

Because the data clearly point toward a downregulation of fatty acid *β*-oxidation in the absence of ANT2 in the RPTCs, whereas the cellular ATP levels appear to be elevated, we next examined another ATP-generating pathway—glycolysis. Despite the extensive glucose entry into the RPTCs (180 g/d), these cells are normally tuned to gluconeogenesis, rather than glycolysis.^14^ Although not reported in RPTCs, enhanced glycolysis in podocytes has been recently found to protect the kidney from the accumulation of toxic metabolites and to improve mitochondrial function.^36^ Therefore, we next assessed systemic nutrient utilization in *RPTC-ANT2*^−/−^ mice and their WT controls fed with a HFD, and while no changes in fat oxidation were found in these mice (Figure 4, G and H), a significant increase in carbohydrate oxidation was measured (Figure 4, I and J). Moreover, increased circulating and urinary levels of lactate were found in these animals (Figure 4, K and L), suggesting enhanced glucose utilization by the kidney. These *in vivo* findings along with our results showing that the glucose-to-lactate ratio in the media of primary RPTCs, extracted from STD-fed mice, was significantly lower in cells lacking ANT2 than their WT counterparts (Figure 4M) indicated enhanced glycolysis in these cells. Indeed, glycolytic Seahorse analysis of ANT2-null RPTCs revealed increases in glycolysis, glycolytic capacity, and reserve (Figure 4, N–Q). Taken together, these findings suggest that elimination of ANT2 in RPTCs rewires their metabolic nutrient utilization fate toward glycolysis.

**Figure 5:**
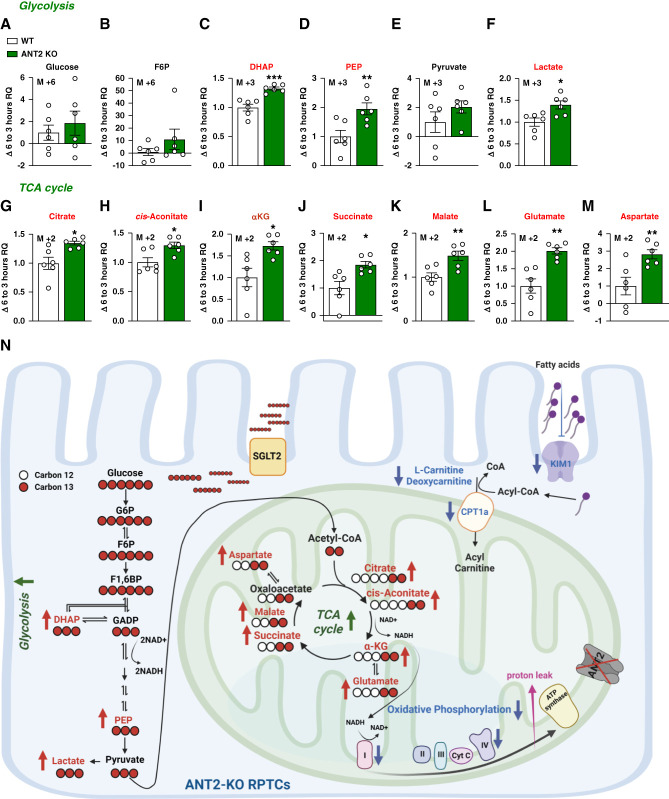
**Increased glycolysis and TCA cycle in ANT2-deleted RPTCs.** A [^13^C]-glucose tracing chase experiment was conducted on primary mouse RPTCs isolated from both STD-fed *RPTC-ANT2*^−/−^ and WT mice and exposed to fatty acid flux for 3 and 6 hours. The alterations (delta) in the incorporation of [^13^C] into labeled metabolites were assessed dynamically from 3 to 6 hours. (A–F) Normalized levels of cellular glycolysis-related metabolites: glucose (A), F6P (B), DHAP (C), PEP (D), pyruvate (E), and lactate (F) (*n*=6 biological replicates per group). (G–L) Normalized levels of cellular TCA cycle–related metabolites: citrate (G), cis-Aconitate (H), α-ketoglutarate (I), succinate (J), malate (K), glutamate (L), and aspartate (M) (*n*=6 biological replicates per group). (N) Graphical representation of intracellular metabolic utilization of glucose in ANT2-depleted RPTCs. The data represent mean±SEM. *P < 0.05, **P < 0.01 or ***P < 0.001 versus WT by Student *t* test. *α*KG, *α*-ketoglutarate; DHAP, dihydroxyacetone phosphate; F6P, fructose 6 phosphate; PEP, phosphoenolpyruvate; TCA, tricarboxylic acid.

To further verify this conclusion, we conducted a large-scale glucose-tracing metabolomics analysis on primary mouse RPTCs collected from both WT and *RPTC-*ANT2^−/−^ mice, which were fed a STD. Subsequently, these cells were exposed to O:P (2:1, 0.1 mM) for 3 and 6 hours, and then, the dynamic changes in the incorporation of [^13^C] into the labeled metabolites were assessed. The analysis revealed increased accumulation velocity of the glycolytic by-products dihydroxyacetone phosphate (DHAP) (Figure 5C), phosphoenolpyruvate (PEP) (Figure 5D), and lactate (Figure 5F) in the absence of ANT2, supporting increased glycolysis in the absence of ANT2. Furthermore, we found increased accumulation of the tricarboxylic acid (TCA) cycle metabolites citrate (Figure 5G), cis-Aconitate (Figure 5H), α-ketoglutarate (Figure 5I), succinate (Figure 5J), malate (Figure 5K), glutamate (Figure 5L), and aspartate (Figure 5M) in ANT2-depleted RPTCs, indicating increased mitochondrial function despite the limited fatty acid β-oxidation (Figure 5N), supporting our Seahorse results.

### RPTC-Induced Glycolysis in ANT2-Null Cells is Controlled by ANT4

**Figure 6:**
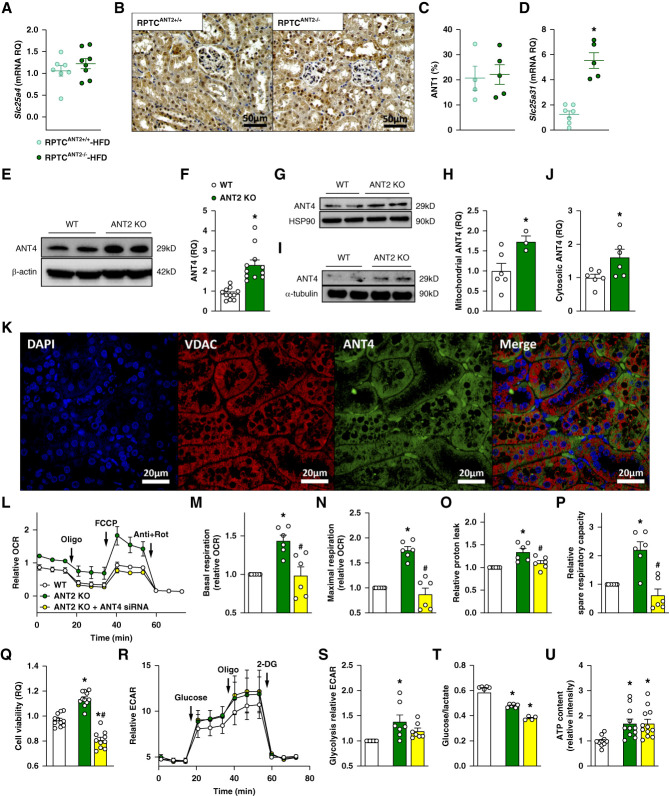
**The lack of RPTC-ANT2 is compensated by the overexpression of ANT4.** (A–C) Gene (A) and quantified immunohistochemical protein expression (B and C) of ANT1 (*Slc25a4*) in *RPTC-ANT2*^−/−^ mice and their WT controls fed with a HFD (*n*=4–8 mice per group). (D–F) Gene (D) and quantified protein levels measured by Western blotting (E and F) of ANT4 (*Slc25a31*) *RPTC-ANT2*^−/−^ mice and their WT controls fed with a HFD (D) or primary mouse RPTCs collected from STD-fed *RPTC-ANT2*^−/−^ and WT mice (E and F) (*n*=5–7 samples per group). (G–J) ANT4 protein expression was quantified by Western blotting in the mitochondrial (G and H) and cytosolic (I and J) fractions of primary mouse RPTCs isolated from STD-fed *RPTC-ANT2*^−/−^ and WT mice (*n*=3–6 biological replicates per group). The data represent mean±SEM. *In vitro*: *P < 0.05 versus WT by Student *t* test. *In vivo*: *P < 0.05 versus RPTC^ANT2+/+^-HFD by Student *t* test. (K) Representative double immunostaining of RPTC-ANT2 KO kidneys and their controls for VDAC and ANT4. (L–U) Primary mouse RPTCs, isolated from STD-fed *RPTC-ANT2*^−/−^ and WT mice, were transfected with a siRNA against ANT4 and the following parameters were measured. (L–P) The Seahorse OCR measurement (L) in ANT4 lacking ANT2 KO RPTCs and their WT controls, from which basal respiration (M), maximal respiration (N), proton leak (O), and spare respiratory capacity (P) were calculated (*n*=6 biological replicates per group). (Q) XTT staining for the cell viability of ANT4 lacking ANT2^−/−^ RPTCs and their WT controls (*n*=10–11 biological replicates per group). (R and S) The Seahorse ECAR measurement (R) in ANT4 lacking ANT2^−/−^ RPTCs and their WT controls, from which glycolysis (S) was calculated (*n*=7 biological replicates per group). (T) Biochemical measurements of the glucose-to-lactate ratio in ANT4 lacking ANT2^−/−^ RPTCs and their WT controls (*n*=4–6 biological replicates per group). (U) Cellular ATP content measured in ANT4 lacking ANT2^−/−^ RPTCs and their controls (*n*=10–12 biological replicates per group). The data represent mean±SEM. *P < 0.05 versus WT, #*P* < 0.05 versus ANT2^−/−^ by one-way ANOVA. siRNA, small interfering RNA; XTT, 2,3-bis-(2-methoxy-4-nitro-5-sulfophenyl)-2h-tetrazolium-5-carboxanilide.

To pinpoint the molecular mechanism by which nullification of ANT2 in RPTCs increases glycolysis and improves cellular and mitochondrial homeostasis, we assessed whether ANT2 deletion is compensated by either ANT1 or ANT4. In fact, both isoforms of ANTs have been linked to glycolysis in cardiac cells^37^ and the testis,^38^ respectively. Whereas the gene and protein expression of ANT1 did not change in ANT2-null RPTCs (Figure 6, A–C), a six-fold increase in the ANT4 gene expression levels and more than two-fold increase in its protein expression levels were found in HFD-fed *RPTC-ANT2*^−/−^ renal cortices and isolated ANT2^−/−^ RPTCs, respectively (Figure 6, D–F). This increase in ANT4 expression was found in both the mitochondrial (Figure 6, G and H) and cytoplasmic (Figure 6, I and J) fractions (Supplemental Figure 10, A and B) of ANT2-null RPTCs. Furthermore, immunofluorescent double staining for ANT4 and VDAC1 (a mitochondrial marker) revealed that ANT4 is not solely present in the mitochondria, but more likely in the cytosol (Figure 6K).

Because ANT4 is predominantly expressed in the testis,^39^ we next determined whether its presence in the mitochondrial and cytosol fractions of RPTCs play any role in regulating mitochondrial homeostasis and cellular function by knocking it down (KD) in primary mouse *RPTC-ANT2*^−/−^ using small interfering RNA (Supplemental Figure 10, C). Our findings show that ANT4 KD (in the absence of ANT2) abolished the increased basal and maximal respiration, proton leakage, and spare respiratory capacity, observed earlier in the absence of ANT2 (Figure 6, L–P). In addition, the increased cell viability of *RPTC-ANT2*^−/−^ was significantly reduced by ANT4 KD (Figure 6Q). Further glycolytic analysis revealed no change neither in glycolytic rate (Figure 6, R–T), nor in cellular ATP content (Figure 6U).

To further verify the renal role of ANT2/ANT4 *in vivo*, we treated chronically obese HFD-fed WT mice with a low dose of carboxyatractyloside (CATR), which inhibits all the isoforms of ANT, and found no improvements in any of the markers related to kidney dysfunction, morphology, lipotoxicity, and fibrosis (Supplemental Figure 5, A–K). In addition, no changes in the ATP levels were found in primary ANT2-null RPTCs treated acutely with CATR (Supplemental Figure 5L). Collectively, these findings suggest that ANT4 mediates the increased ATP content by aerobic glycolysis and improves mitochondrial integrity, cellular homeostasis, and kidney function in the absence of ANT2 in RPTCs.

### Weight-Independent Improvements in Whole-Body Metabolism in *RPTC-ANT2*^−/−^ Mice

**Figure 7:**
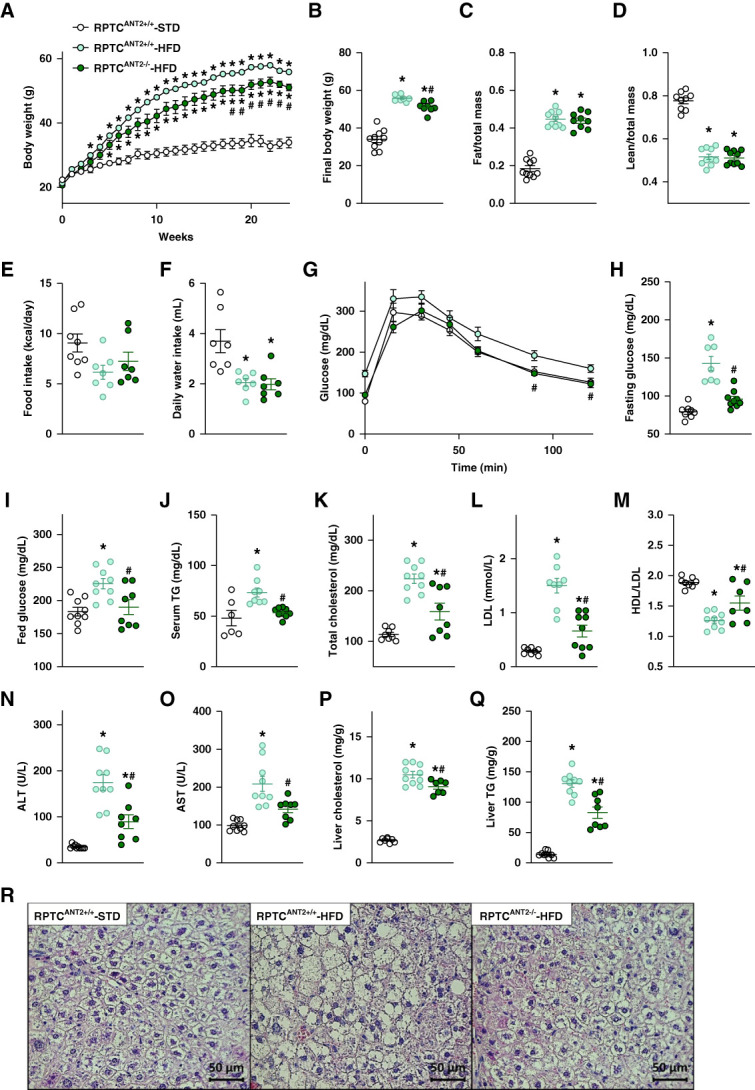
**Weight-independent improvements in whole-body metabolism in obese *RPTC-ANT2***^**−/−**^ **mice.** *RPTC-ANT2*^−/−^ mice and their WT littermate controls were fed either a STD or a HFD for 24 weeks. (A and B) Weekly body weight (A) and final (B) measurements of *RPTC-ANT2*^−/−^ animals and their WT littermate controls fed either STD or HFD (*n*=8–9 mice per group). (C and D) MRI measurements of relative fat (C) and lean (D) masses in *RPTC-ANT2*^−/−^ animals and their WT littermate controls fed either STD or HFD (*n*=9–10 mice per group). (E and F) Daily food (E) and water (F) intakes of *RPTC-ANT2*^−/−^ animals and their WT littermate controls fed either STD or HFD (*n*=7–8 mice per group). (G) Glucose tolerance test (*n*=8–9 mice per group). (H–O) Biochemical measurements of fasting blood glucose (H), fed serum glucose (I), TGs (J), total cholesterol (K), LDL (L), the HDL-to-LDL ratio (M), ALT (N), and AST (O) (*n*=6–10 mice per group). (P and Q) Biochemical measurements of hepatic cholesterol (P) and TG (Q) content (*n*=7–10 mice per group). (R) Representative liver tissue H&E staining. The data represent mean±SEM. *P < 0.05 versus RPTC^ANT2+/+^-STD, #*P* < 0.05 versus RPTC^ANT2+/+^ HFD by one-way ANOVA or two-way ANOVA for time-dependent measurements. H&E, hematoxylin & eosin.

After observing the protective effects of eliminating ANT2 in RPTCs against obesity-induced CKD, we proceeded to assess its influence on systemic metabolism. Both animal cohorts, encompassing WT and *RPTC-ANT2*^−/−^ mice, displayed substantial weight gain when subjected to an HFD, reaching an average final weight exceeding 55 grams. Notably, the obese *RPTC-ANT2*^−/−^ mice exhibited a marginally leaner phenotype compared with their WT counterparts (Figure 7, A and B). Surprisingly, no significant differences were found in fat or lean masses (Figure 7, C and D), food or water consumption (Figure 7, E and F), bone mass or length (Supplemental Figure 6), as well as whole-body energy expenditure and activity profiles (Supplemental Figure 7). Similar findings were also recorded in female HFD-fed *RPTC-ANT2*^−/−^ mice and their obese littermate WT controls (Supplemental Figure 8). These findings may suggest other mechanisms contributing to the lower body weight gain of *RPTC-ANT2*^−/−^ mice.

As noted earlier, obesity-induced CKD results in mitochondrial dysfunction that diminishes RPTC function, leading to altered renal glucose and lipid metabolic profiles, which in turn may contribute to systemic metabolic alterations. In addition to their role in nutrient reabsorption, RPTCs may affect systemic homeostasis through multimodular interactions with other tissues. Therefore, with the noticeable slight but significant reduction in the body weight gain of HFD-fed *RPTC-ANT2*^−/−^ mice, we next assessed whether nullification of RPTC ANT2 affected any other metabolic parameters irrespective of weight loss. Surprisingly, glucose intolerance and circulating glucose levels under fasted and fed conditions were significantly improved in the obese *RPTC-ANT2*^−/−^ mice (Figure 7, G–I). Furthermore, dyslipidemia, evident by hypertriglyceridemia, hypercholesteremia, increased LDL-cholesterol levels, and a reduced HDL-to-LDL ratio were completely normalized in obese *RPTC-ANT2*^−/−^ mice (Figure 7, J–M). Finally, HFD-induced liver injury and hepatic steatosis in obese WT animals were ameliorated in HFD-fed *RPTC-ANT2*^−/−^ mice (Figure 7, N–R). Collectively, these findings suggest that loss of ANT2 in RPTCs may not only have a positive, local effect on kidney function but also mediates improvements in systemic metabolism. Remarkably, these improvements were completely replicated by chronically administering 1 mg/kg CATR to obese HFD-fed WT animals (Supplemental Figure 9), implying that ANT4 in RPTCs is unlikely to mediate these systemic changes.

## Discussion

A large body of evidence supports the notion that obesity leads to kidney dysfunction, associated with deterioration in mitochondrial function, integrity, biogenesis, architecture, and respiration. In this study, we revealed that ANT2, whose function is tightly coupled to OXPHOS, plays a major role in the development of obesity-induced CKD. Specific deletion of ANT2 from the mitochondria-rich RPTCs protects the mice from renal injury, lipotoxicity, inflammation, and fibrosis. Unbiased transcriptomics, proteomics, and metabolomics analyses revealed that without ANT2, RPTCs underwent metabolic rewiring, from utilization of fatty acids through the *β*-oxidation pathway toward increased aerobic glycolysis to fulfill the energy needs and to maintain cellular integrity and viability. These changes are most likely mediated by the compensation of ANT4 for the loss of ANT2. Overall, targeting RPTC-ANT2 or its upstream/downstream pathways could be a novel therapeutic approach for treating obesity-induced CKD.

Lipotoxic conditions has been shown to reduce ANT2 abundance in various tissues.^26,40^ Our findings support this, showing downregulation of ANT2 in the kidney and more specifically in RPTCs after exposure to fatty acids. Because ANT2, which regulates cellular ATP levels, was downregulated in the kidneys of HFD-fed animals, our initial assumption was that the complete absence of ANT2 under lipotoxic conditions should result in kidney damage. However, contrary to our initial hypothesis, complete absence of ANT2 in RPTCs under lipotoxic conditions resulted in complete protection from obesity-related renal damage. Deletion of ANT2 in RPTCs preserved kidney function and prevented renal lipotoxicity, fibrosis, and morphological changes of the glomeruli, which are strongly associated with tubular damage.^41–43^ These findings suggest that downregulation of renal ANT2 in HFD-fed mice or in RPTCs exposed to lipotoxic conditions represents a failed organismal adaptation aimed to prevent injury. Whereas global deletion of ANT2 is embryonic lethal,^24^ our cell-specific approach and novel findings support earlier studies showing improved cellular function in hepatocytes, adipocytes, and macrophages,^25–27,40^ suggesting that targeting ANT2 could prevent/treat renal complications.

Mitochondrial dysfunction is common in kidney diseases,^44–46^ and because the kidney mostly relies on OXPHOS for ATP synthesis,^47^ mitochondrial homeostasis is essential for optimal kidney function. Given the important role of ANT2, one would expect that deleting it from RPTCs would severely impair cellular respiration by reducing the abundance of ADP required for ATP synthesis. However, surprisingly, ANT2 deletion from RPTCs increased whole cellular ATP content and improved mitochondrial function, an effect that was not associated with alterations in mitogenesis or mitochondrial architecture. Similarly, Parodi-Rullán and colleagues showed that ANT1 silencing in cardiac cells elevates ATP levels^37^; this contradicts other reports, showing no change in ATP content in human embryonic kidney-293 cells,^48^ reduced ADP/ATP exchange ratio in hepatocytes,^25^ or decreased ATP production in bronchial epithelial cells^49^ after the deletion of ANT2. Because knockdown of ANT2 was performed in different cell types, it could probably explain these conflicting results.

Previous studies have shown that whereas ANT1 is responsible for the basal respiratory activity of the mitochondria, ANT2 exclusively mediates fatty acid–induced uncoupled respiration.^26,28^ Such uncoupling may lead to a state of relative hypoxia and the induction of an HIF-1*α* transcriptional program.^26^ Increased OXPHOS activity in ANT2-deleted macrophages has been recently reported in macrophages and adipose tissue.^40^ Interestingly, our transcriptomics and proteomics analyses of kidney samples collected from *RPTC-ANT2*^−/−^ mice and their WT littermates revealed a robust reduction in OXPHOS in the null mice, which conflicts with increased mitochondrial OCR. However, the observed compensatory increased activity of complex IV^50^ could explain the increased mitochondrial OCR. The decreased OXPHOS has been further confirmed here by the decreased CPT1a expression and levels of carnitines required for its proper function as well as a reduction in AMPK and SIRT1 activities, which are the main regulators of fatty acid *β*-oxidation–related OXPHOS. Interestingly, our proteomics analysis revealed a reduction in mitochondrial fatty acid *β*-oxidation coupled with an increase in peroxisomal FAO. It has been shown that peroxisomal FAO can act as a compensatory mechanism at times when no fatty acids are being transported into the mitochondria.^51,52^ In fact, increased paroxysmal *β*-oxidation has been reported to ameliorate mitochondrial fitness and protect these organelles against oxidative insult.^52,53^ This unexpected outcome suggests an intriguing metabolic adaptation wherein the utilization of fatty acids through their renal uptake and mitochondrial oxidation in RPTCs is diminished while paradoxically, the mitochondria remain functionally robust and healthy despite lipotoxic conditions. Specifically, we found that nullification of ANT2 in RPTCs results in a robust reduction in fatty acid uptake, most likely mediated by reduced expression of KIM-1. Our findings suggesting increased clearance of free fatty acid by the kidney in *RPTC-ANT2*^−/−^ mice further support the recent reported role of KIM-1 in mediating fatty acid uptake by RPTCs.^54^ Taken together, our findings portray a unique metabolic state, in which mitochondria maintain their functionality and health in lipotoxic conditions despite reduced utilization of fatty acids via renal uptake and beta-oxidation.

Normally, RPTCs use *β*-oxidation for producing copious amounts of energy for their highly demanding reabsorption tasks^12–14^; however, the increased cellular ATP levels in the absence of ANT2 could result from an aerobic glycolytic compensation. Indeed, a careful analysis of whole-body energy utilization during the day/night transition revealed increased carbohydrate levels rather than FAO in HFD-fed *RPTC-ANT2*^−/−^ animals, which was coupled with increased levels of circulating and urinary lactate, a dead-end product of glycolysis. These findings were further verified in isolated RPTCs, showing a reduced glucose-to-lactate ratio, an elevated extracellular acidification rate, and an increased glycolytic capacity and reserve in the ANT2 null cells. These data were further confirmed by metabolomics flux analysis, demonstrating increased glycolysis and TCA cycle, which together could explain the elevated cellular ATP levels. While these findings support increased glucose utilization by the mitochondria, one cannot negate the role of other unexplored mitochondrial fuels to the observed cellular and mitochondrial phenotypes. Nevertheless, similar findings were reported in other studies.^30,49^ In fact, many studies have described a metabolic rewire from *β*-oxidation to aerobic glycolysis in the kidney at times of metabolic stress.^55^ One possible mechanism for this metabolic shift toward glycolysis is by changes in the activation state of pyruvate dehydrogenase complex.^49,52^ However, further work needs to confirm its role in the metabolic adaptation of ANT2-deleted RPTCs. Interestingly, data from human metabolomics and transcriptomic databases have demonstrated an upregulation of aerobic glycolysis in patients with CKD.^50,51^ The observed elevation in lactate levels in our model may raise a concern because ATP production through lactate is considered an inefficient energy pathway compared with the conversion from pyruvate to acetyl-CoA. Although this shift might initially conserve oxygen and ATP within cells, over time, it could result in mitochondrial dysfunction, reduced energy generation, and kidney damage. Notably, increased lactate production in the kidney has been linked to diabetic CKD pathogenesis, and elevated urinary lactate levels might serve as a potential biomarker indicating the risk of CKD progression.^56^ While our data suggest a positive renal response to this metabolic shift, its underlying cause remains unknown. Nevertheless, we suggest that ANT2 depletion in RPTCs under lipotoxic conditions may trigger a long-lasting protective metabolic rewiring toward glycolysis by a compensatory upregulation of ANT4 expression, allowing these cells to maintain energy production, which in turn promotes the preservation of renal function and ameliorates obesity-induced CKD.

In our search for the mechanism underlying the metabolic shift in ANT2-null RPTCs, we found an unexpected upregulation of another ANT paralog—ANT4. ANT4 is a unique protein among the ANT members because of its low sequence identity, and the fact that it is exclusively expressed in the testis and sperm, and that it is methylated everywhere else.^57,58^ Although it has been shown that humans express the ANT4 gene to a certain degree in several tissues, including the kidney,^59^ its presence in mouse tissues has been shown to be strictly restricted to the male reproductive system^58^ and female ovaries at a certain developmental stage.^60^ In fact, according to the single-cell RNA-seq data found at the Humphreys/Susztak Lab database, the expression of *Slc25a31*, the gene encoding ANT4 protein, in mouse kidney is barely discernible. Although other studies in which ANT2 was depleted in different cells did not find any compensatory increase in other ANT paralogs,^25,48^ we show for the first time a robust increase in ANT4, but not in ANT1, in ANT2-deleted RPTCs. Our data may, therefore, suggest the novel idea of a reciprocal interaction between ANT2 and ANT4 in the kidney, particularly in conditions involving the deletion/reduction of ANT2. Interestingly, such a reciprocal interaction has been suggested earlier, likely because of their distinct chromosomal locations in mammals, with ANT2, found on the X chromosome, being subject to transcriptional repression during male meiosis, while ANT4, located on autosomes, potentially compensates for this loss of ANT2 function in this context.^61^ In fact, the unique function of ANT4 in glycolysis^62^ caught our attention, and we found, similarly to others,^62^ that its presence is not solely confined to the mitochondria, but rather it is present and elevated also in the cytosol of ANT2 KO RPTCs. Reducing its expression not only decreased the elevated respiration in ANT2-deleted RPTCs but also reduced their viability. Of note, the ANT4 knockdown did not affect the glycolytic parameters, presumably because the absence of both ANT2 and ANT4 is lethal for the cells. Blocking all ANTs (with CATR) completely abolished the beneficial effects of ANT2 knockout, further supporting the relevance of ANT4 in preserving kidney function in obesity. Altogether, these data suggest that ANT4 compensates for the loss of ANT2 and is responsible for the metabolic shift in these cells; therefore, deletion/inhibition of both ANTs would impair cellular and kidney function.

Another very surprising effect that our study revealed is that nullification of ANT2 specifically in RPTCs resulted in much greater systemic metabolic improvements in the null mice fed with an HFD. These benefits included restoration of glucose homeostasis and amelioration of dyslipidemia, hepatic steatosis, and liver injury in obese *RPTC-ANT2*^−/−^ mice. Interestingly, cell-specific knockout of ANT2 in hepatocytes and adipocytes similarly induced systemic beneficial metabolic effects,^25,27^ raising the intriguing question of whether all these mouse models share a similar mechanism that mediates these effects or whether nullification of ANT2 in each cell triggers a distinct cellular pathway that somehow may lead to a unique metabolic phenotype. Especially exciting findings were the lack of HFD-induced liver steatosis in the absence of ANT2 in RPTCs, suggesting a role for ANT2 in the barely explored kidney-to-liver axis crosstalk. Because pharmacologically blocking all isoforms of ANT by using CATR in HFD-fed WT mice replicated these metabolic improvements, our data suggest that ANT4 in RPTCs does not mediate these positive metabolic changes. Further studies would need to assess whether one or more specific renal soluble mediators affect hepatic function and metabolism in the presence or absence of ANT2.

In conclusion, our findings revealed RPTC-ANT2 as a major player in the development of obesity-induced CKD. Its nullification from RPTCs exerts a metabolic shift toward aerobic glycolysis and enables mitochondrial protection and survival. Our work also suggests that ANT4 mediates the metabolic transition in RPTCs and that ANT2 in RPTCs may also (indirectly) affect systemic metabolism. Taken together, our work highlights the therapeutic potential of targeting ANT2 (and/or ANT4) for the treatment of obesity-induced CKD.

## Supplementary Material

## Data Availability

Data related to transcriptomic, proteomic, or metabolomic data (per previous question). The mass spectrometry proteomics data have been deposited to the ProteomeXchange Consortium via the PRIDE partner repository with the dataset identifier PXD042128.

## Supplemental Material

Supplemental Methods and References

Supplemental Figure 1. Generating RPTC-specific ANT2-null mice.

Supplemental Figure 2. Effect of RPTC-ANT2 nullification on transcriptome-level cellular and organelle functions.

Supplemental Figure 3. Effect of ANT2 nullification on oxidative and nitrosative stress in primary mouse RPTCs.

Supplemental Figure 4. Effect of ANT2 nullification in RPTCs on mitochondrial characteristics.

Supplemental Figure 5. Carboxyatractyloside (CATR) treatment does not replicate the positive renal effect of RPTC-ANT2 nullification in high-fat diet (HFD)-fed mice.

Supplemental Figure 6. RPTC ANT2 deletion does not affect bone morphology.

Supplemental Figure 7. RPTC ANT2 nullification does not affect whole-body metabolism or activity.

Supplemental Figure 8. Female RPTC-ANT2 KO mice display a similar metabolic phenotype to that of male animals.

Supplemental Figure 9. Carboxyatractyloside (CATR) treatment improves systemic metabolism in high-fat diet (HFD)-fed WT mice similarly to RPTC-ANT2 genetic ablation.

Supplemental Figure 10. Validation of cell fractionation and siRNA transfection.

Supplemental Excel Sheet 1. Source Data A: provides the original raw data of the NanoString transcriptomics results, including the expression levels of all the genes analyzed in the assay.

Supplemental Excel Sheet 2. Source Data B: offers information on the raw data for each panel in the Figures and Supplementary Figures.
