# Supplementary material for: Renal Mitochondrial ATP Transporter Ablation Ameliorates Obesity-Induced CKD
Source: J Am Soc Nephrol. 2024 Jan 11;35(3):281–98. doi: 10.1681/ASN.0000000000000294 (PMC10914206; doi:10.1681/ASN.0000000000000294)
Supplement: Supplementary file 1 [file jasn-35-281-s001.pdf]

Inventory of Supporting Information

Renal Mitochondrial ATP Transporter Ablation Ameliorates Obesity-induced Chronic Kidney Disease

Anna Permyakova[1], Sharleen Hamad[1], Liad Hinden[1], Saja Baraghithy[1], Aviram Kogot-Levin[2], Omri Yosef[3], Ori Shalev[4], Manish Kumar Tripathi[5], Haitham Amal[5], Abhishek Basu[6], Muhammad Arif[6, 7], Resat Cinar[6], George Kunos[8], Michael Berger[3], Gil Leibowitz[2], and Joseph Tam[1*].

[*]Correspondence to: yossi.tam@mail.huji.ac.il

Supplementary Methods and References

Supplementary Figure 1. Generating RPTC-specific ANT2-null mice.

Supplementary Figure 2. Impact of RPTC-ANT2 nullification on transcriptome-level cellular and organelle functions.

Supplementary Figure 3. Effect of ANT2 nullification on oxidative and nitrosative stress in primary mouse RPTCs.

Supplementary Figure 4. Impact of ANT2 nullification in RPTCs on mitochondrial characteristics.

Supplementary Figure 5. Carboxyatractyloside (CATR) treatment does not replicate the positive renal effect of RPTC-ANT2 nullification in high-fat diet (HFD)-fed mice.

Supplementary Figure 6. RPTC ANT2 deletion does not affect bone morphology.

Supplementary Figure 7. RPTC ANT2 nullification does not affect whole-body metabolism or activity.

Supplementary Figure 8. Female RPTC-ANT2 KO mice display a similar metabolic phenotype to that of male animals.

Supplementary Figure 9. Carboxyatractyloside (CATR) treatment improves systemic metabolism in high-fat diet (HFD) fed WT mice similarly to RPTC-ANT2 genetic ablation.

Supplementary Figure 10. Validation of cell fractionation and siRNA transfection.

Supplemental Excel Sheet 1: Source Data A: Provides the original raw data of the NanoString transcriptomics results, including the expression levels of all the genes analyzed in the assay.

Supplemental Excel Sheet 2: Source Data B: Offers information on the raw data for each panel in the Figures and Supplementary Figures

Supplementary Methods

Animals

The experimental protocols were approved by the Institutional Animal Care and Use Committee of the Hebrew University of Jerusalem (AAALAC accreditation #1285; Ethic approval number MD-19-15783-4 and MD-22-16932-2), and are in compliance with the ARRIVE guidelines. All animals used in this study were housed under specific pathogen-free (SPF) conditions, up to five per cage, in standard plastic cages with natural soft sawdust as bedding. The animals were maintained under a controlled temperature of 22–24°C, humidity at 55 ± 5%, and alternating 12-hour light/dark cycles, and provided with food and water *ad libitum*. A novel RPTC-ANT2 null mouse strain was generated using the Cre-Lox recombination system. Briefly, a C57BL/6J mouse, carrying *Cre* recombinase under the promotor of a *Sglt2* gene (iL1-sglt2-Cre), which specifically expresses *Cre* recombinase in the brush border membrane of the S1 segment of the proximal tubule[1] was paired with a C57BL/6J mouse, which systemically expresses LoxP sites 131 bp upstream of the second exon and 189 bp downstream of the third exon of the *Slc25a5* gene[2] (Jackson laboratory, Strain #029482). The offspring of this pairing were identified as either RPTC-ANT2$^{-/-}$ or their WT littermates at the gene and protein levels. Mice were genotyped by PCR. For verifying the recombination of the ANT2 gene (*Slc25a5)*, the primers 5'-GCAGCCATCTCCAAGACAG - 3', and 5'- TGATCTCATCATACAAGACAAGCA - 3' were used. ANT2 null samples produced a band of 201bp, whereas WT samples produced a band of 829bp. To identify *Cre* recombinase, the following primers were used: 5'-AGCCTGTTTTGCACGTTCACC-3', 5'-GGTTTCCCGCAGAACCTGAA-3'. To identify the *LoxP* sites, the following primers were used: 30584 5'- CTTCTGATGTGCTGCTCAA-3', 30585 5'- GGAAGAAGGCAATAGGGACA-3'.

To generate RPTC-ANT2 null animals with green fluorescent mitochondria, we crossed RPTC-ANT2$^{-/-}$ mouse and PhAM^{floxed} transgenic mouse with a mitochondrial-specific version of Dendra2 green/red photoswitchable monomeric fluorescent protein[3] (Jackson Laboratory, Strain # 018385).

To validate the presence of ANT2 protein in RPTCs, SGLT2-YFP mice were used[4]. These animals, carrying the YFP gene with a floxed STOP codon, were crossed with animals expressing the Cre recombinase under the promotor of *Sglt2*. The results of this breeding were mice that express YFP specifically in the RPTCs (Sglt2-Cre;Rosa26-YFP reporter mice).

Experimental protocol

To generate diet-induced obesity, six-week-old male and female RPTC-ANT2$^{-/-}$ mice and their WT littermates as well as Sglt2-Cre;Rosa26-YFP reporter mice were fed either a high-fat diet (HFD; Research Diet, D12492; 60% of calories from fat, 20% from protein and 20% from carbohydrates) or a standard diet (STD; 14% fat, 24% protein, 62% carbohydrates; NIH-31 rodent diet) for 24 weeks.

As per the pharmacological model of ANT blockade, six-week-old male C57BL/6J mice were fed with a HFD for 28 weeks. During the last 4 weeks of the experiment, the mice received a daily 1 mg/kg i.p. injection of the ANT inhibitor, carboxyatractyloside (CATR; Cat#3202, AvaChem Scientific (USA)[5] or vehicle (saline).

Under both experimental conditions, the body weight was monitored each week (during the diet regimen) or daily (during the pharmacological treatment). A 24-hour urine output was measured and collected using the CCS2000 Chiller System (Hatteras Instruments). Body composition was determined by EchoMRI-100H™ (Echo Medical Systems). Mice were

euthanized by a cervical dislocation under anesthesia; trunk blood, kidneys, pancreas, liver, brain, and bones were collected and samples were either snap-frozen or fixed in buffered 4% formalin for further analyses.

Cell culture

Primary mouse RPTCs from any strain (RPTC-ANT2$^{-/-}$, DENDRA RPTC-ANT2$^{-/-}$ and their littermate controls) utilized in this work were used following their ex-vivo extraction by using the following protocol: Mouse kidney cortices were dissociated into single cells using 0.7 mg/mL collagenase/dispase (Sigma-Aldrich; Cat# 10269638001) in Hanks' Balanced Salt Solution (HBSS), followed by vortex in Gentle MACs Dissociator (MACS Miltenyi Biotec) program Multi_E_02. Red blood cells were removed using RBC Lysis solution (Sartorius; Cat #01-888-1B). RPTCs were purified using a low speed (100 × g) centrifugation step, and then cultured in a REGM BulletKit medium (Lonza; Cat #CC-3191 & #CC-4127), containing 5.5 mM glucose on collagen-coated 6-well plates.

HEK-293 (Cat# CRL-1573, ATCC) and HK-2 (Cat# CRL-2190, ATCC) cells were cultured in high-glucose (HG)-DMEM and low-glucose (LG)-DMEM, respectively, supplemented with a fetal bovine serum (10% and 5%, respectively) and 1% of 100 IU/mL penicillin/streptomycin, L-Glutamine 29.2 mg/mL, and sodium pyruvate 11 mg/mL (03-031-1B, 03-020-1B and 03-042-1B, respectively; Sartorius, Israel) at 37°C in a humid atmosphere with 5% CO_2.

To test the effect of a lipotoxic environment on ANT2 expression, primary mouse RPTCs or HK-2 cells were cultured overnight with serum-free REGM or LG-DMEM, respectively,

supplemented with 0.1% BSA. The cells were then incubated for 24-48 h with an oleate:palmitate (O:P, 2:1) mixture[6,7] (0.1 mM or 0.25 mM, respectively).

To test the effect of a pharmacological ANT inhibition, primary mouse ANT2-deleted RPTCs were cultured overnight with a serum-free REGM supplemented with 0.1% BSA. The cells were then incubated for 24 h with 0.1 μM CATR.

The XTT-based Cell Proliferation Kit (Cat# 20-300-1000, Sartorius) was used to determine the RPTC viability. The cells were stained according to the manufacturer's instructions and the absorbance was measured following 4 hours of incubation.

Multi-parameter metabolic assessment

Mouse activity and respirometry parameters as well as daily water and food intakes were assessed by using the Promethion High-Definition Behavioral Phenotyping System (Sable Instruments, Inc., Las Vegas, NV, USA) as described previously by us[8]. The analysis was done on data collected after a 24-hour period or a 4-hour period in the dark-to-light transition.

Bone mass and structure analysis by μCT

Briefly, femora were examined using a μCT system as described previously[9] (μCT 40; Scanco Medical AG) at a 10-μm isotropic resolution, with an X-ray tube potential of 70kVp, intensity of 114 μA, and an integration time of 200 ms. In the femora, trabecular bone parameters were measured in the distal metaphyseal segment, extending 3 mm proximally from the proximal tip of the primary spongiosa. Cortical bone parameters were determined in a diaphyseal segment extending 1 mm distally from the midpoint between the femoral ends.

Electron Microscopy

Kidney slices (3 mm) were fixed overnight in 2% paraformaldehyde and 2.5% glutaraldeyde in 0.1 M cacodylate buffer (pH 7.4) at room temperature, and then washed four times in cacodylate buffer. Tissue slices were stained with 1% osmium tetroxide, 1.5% potassium ferricyanide in 0.1 M cacodylate buffer for 1 hour, washed four times in cacodylate buffer, and dehydrated. Following dehydration, slices were infiltrated with increasing concentrations of Agar 100 resin in propylene oxide, consisting of 25%, 50%, 75%, and 100% resin for 16 hours each, and were then embedded in fresh resin, and allowed to polymerize at 60°C for 48 hours. Embedded tissues in blocks were sectioned with a diamond knife on a Leica Reichert Ultracut S microtome, and ultrathin sections (80 nm) were collected onto 200 Mesh, carbon–formvar-coated copper grids. The sections on grids were sequentially stained with uranyl acetate and lead citrate for 10 minutes each and were viewed with Tecnai 12 TEM 100 kV (Phillips, Eindhoven, The Netherlands) equipped with a MegaView II CCD camera and Analysis version 3.0 software (SoftImaging System GmbH, Münstar, Germany). The images in .tiff format were analyzed using Adobe Photoshop C3S software.

Blood, kidney, and urine biochemistry

Urine albumin (Bethyl Laboratories; Cat# E99-134) and Clusterin (Abcam; Cat# ab199079) were measured by ELISAs. Serum glucose, lactate, alanine transaminase (ALT), aspartate aminotransferase (AST), alkaline phosphatase (ALP), triglycerides (TG), high-density lipoprotein (HDL), low-density lipoprotein (LDL), and total cholesterol, as well as urine creatinine and lactate were determined using the Cobas C-111 bio-analyzer (Roche, Switzerland). Serum and urine free fatty acid content was determined using Free Fatty Acid Assay Kit (Abcam; Cat# ab65341).

Whole kidney proteins were extracted using an Extraction Buffer 5X PTR (Abcam; Cat# ab193970), and the levels of the neutrophil gelatinase-associated lipocalin (NGAL; Abcam; Cat# ab199083), kidney injury marker 1 (KIM-1; Abcam; Cat# ab213477), Cystatin C (Abcam; Cat# ab201280), TIMP metallopeptidase inhibitor 1 (TIMP1; Abcam; Cat# ab196265) and Clusterin (Abcam; Cat# ab199079) were measured using ELISA kits.

Glucose tolerance test (GTT)

Mice that fasted overnight were injected with glucose (1.5 g/kg, ip), followed by a tail blood collection at 0, 15, 30, 45, 60, 90, and 120 minutes. Blood glucose levels were determined using the Elite glucometer (Bayer, Pittsburgh, PA).

Hepatic and renal lipid content

Kidney and liver lipids were extracted as previously described[10] and their TG and cholesterol contents were determined using a Cobas C-111 bio-analyzer (Roche, Switzerland).

Cellular fatty acid uptake

Intracellular lipid uptake was measured by following the protocol reported by Greenspan and colleagues[11] with modification. Briefly, primary mouse RPTCs were incubated in black 96-well plates at a density of 4×10^4 cells per well. Each well was washed twice with 1x PBS and then stained with Nile Red (Sigma–Aldrich; Cat# 19123) and Hoechst by adding a Nile Red/Hoechst mixed solution (1 µg/mL; diluted in 1 × PBS) to the cells for 15 min at 37 °C. The cells were then washed with 1 × PBS, and the fluorescence intensity was measured with a SpectraMax iD3

microplate reader (Molecular Devices) at wavelengths of ex:488/em:550 and ex:350/em:461 nm for Nile Red and Hoechst, respectively. The Nile Red results were normalized Hoechst results.

Histopathology

Paraffin-embedded kidney sections (3 µm) from each mouse were stained with periodic acid–Schiff (PAS) (Abcam; Cat# ab150680), followed by hematoxylin (Abcam; Cat# ab220365), as well as trichrome stain (Abcam; Cat# ab150686). Paraffin-embedded liver sections (4 µm) from each mouse were stained with H&E (Abcam; Cat# ab245880). Kidney and liver images were taken from 10 random 40 × fields with an AxioCam ICc5 color camera mounted on an Axio Scope.A1 light microscope (Zeiss, Germany). The mesangial expansion, glomerular, Bowman's space, and fat vacuolated RPTCs cross-sectional areas were quantified in the kidney in a blinded manner using Adobe Photoshop CS3 software.

Immunostaining

For immunofluorescent assessment of the presence of ANT2 in the RPTCs, kidney sections were double stained with rabbit ANT2 (Cell signaling Technology; Cat# 14671, 1:200) and goat YFP (Abcam; Cat# ab6673), followed by incubation with goat anti-rabbit Cy3 conjugate (Jackson ImmunoReaserch Laboratories; Cat# 111-165-144), anti-goat Cy5 conjugate (Jackson ImmunoReaserch Laboratories; Cat# 805-605-180), and DAPI (KPL; Cat# KP-71-03-01). Stained sections were photographed using a Nikon A1R confocal laser scanning microscope (magnification of ×60).

To examine the cellular location of ANT4 in RPTCs, kidney sections were stained with rabbit ANT4 (Biorbyt; Cat# orb159946, 1:50) and mouse VDAC1/Porin (Abcam; Cat# ab14734, 1:300), followed by incubation with anti-rabbit Cy5 conjugate (Jackson ImmunoReaserch Laboratories; Cat# 711-175-152), anti-mouse Cy3 conjugate (Jackson ImmunoReaserch Laboratories; Cat# 715-165-151), and DAPI (KPL; Cat# KP-71-03-01). Stained sections were photographed using a Nikon AXR confocal laser scanning microscope (magnification of ×100).

For immunohistochemical evaluation, kidney sections were stained with rabbit ANT1 (Abcam; Cat# ab192351) and rabbit phospho-AMPK (Abcam; Cat# ab133448) antibodies, followed by an anti-rabbit HRP conjugate (ImmPRESS™, Vector laboratories). Color was developed after an incubation with 3,3-'Diaminobenzidine (DAB) substrate (ImmPACT DAB Peroxidase (HRP) Substrate, SK-224105, Vector Laboratories), followed by hematoxylin counterstaining. The images were taken from 10 random fields (magnification of ×40) with an AxioCam ICc5 color camera mounted on an Axio Scope.A1 light microscope (Zeiss, Germany). The positive (stained) area for each marker was calculated using color thresholding and measuring the area fractions with Image J software (NIH Public Domain), with a minimum of 5-6 random kidney sections per mouse. Images are presented in the figures, showing the animal with the median value for each group.

Small interfering RNA treatment

Small interfering RNA (siRNA) transfection against ANT4 (Santa Cruz; Cat# sc-105072) was performed in primary mouse RPTCs using the siRNA reagent system (Santa Cruz; Cat# sc-45064), according to the manufacturer's instructions and as verified in **Supplementary Figure 10**.

Flow Cytometry

Primary mouse RPTCs were extracted from DENDRA RPTC-ANT2$^{-/-}$. The cells were maintained in a FACS buffer (PBS containing 2% FBS + 1 mM EDTA). Cells were acquired by a CytoFlex LX machine flow cytometer with CytExpert software (Beckman Coulter, Brea, CA), and analyzed by FCS Express 6 (De Novo Software). Dendra2 intensity was read via 480 laser (525 ±40 Filter), pre-gated on single cells using SSC-A vs SSC-H.

For mitochondrial membrane potential staining, cells were labeled with 50 nM Tetramethylrhodamine, Methyl Ester, Perchlorate (TMRM; Invitrogen, Cat# I34361) in a FACS buffer without EDTA (PBS + 2% FCS) for 30 min at 37 °C. Stained cells were analyzed by a CytoFLEX V2-B4-R2 Flow Cytometer and analyzed by FCS Express 6.

Extracellular flux analysis

The cellular oxygen consumption rate (OCR) and the extracellular acidification rate (ECAR) were measured using an Agilent Seahorse XFe96 Analyzer (Agilent Technologies, USA) as recommended by the manufacturer. ANT2 null, ANT4 KD or WT primary mouse RPTCs were seeded on XF96 collagen-coated cell culture plates at a density of 40 × 10^3 cells/well, and incubated overnight in REGM BulletKit medium (Lonza; Cat #CC-3191 & #CC-4127), containing 5.5 mM glucose at 37 °C with 5% CO_2. On the day of the experiment, cells were switched to DMEM media, pH 7.4 (Agilent Seahorse XF; Cat# 103575-100) supplemented with 5.5 mM glucose. OCR or ECAR were assessed using the Mito Stress Test or Glycolysis Stress Test programs, respectively. In the Mito Stress Test, cells were exposed to 1 µM of oligomycin, carbonyl cyanide-p-trifluoromethoxyphenylhydrazone (FCCP), antimycin, and rotenone to evaluate mitochondrial function. In the Glycolysis Stress Test, cells were exposed to 10 mM

glucose, 1 μM oligomycin, and 50 mM 2-deoxy-d-glucose (2-DG) to assess glycolytic activity. The results were standardized based on cell count and are depicted in the paper relative to the control group. Additionally, in the figures showing OCR and ECAR measurements over time, the data were also normalized to OCR measured after the addition of rotenone and antimycin A, or to ECAR measured after adding 2-DG.

Mitochondrial function assessment

ATP content in primary mouse RPTCs was measured using a luminescent ATP Detection Assay Kit (Abcam; Cat# ab113849). Reactive oxygen species (ROS) in primary mouse RPTCs were measured using the fluorescent DCFDA/H2DCFDA – Cellular ROS Assay Kit (Abcam; Cat# ab113851). The NAH^+/NADH ratio in RPTCs was measured using a colorimetric NAD^+/NADH Assay Kit (Abcam; Cat# ab65348). The nitrosative stress was assessed in the form of measuring nitric oxide (NO) metabolites, nitrate and nitrite, using the Griess Assay Kit, in accordance with the manufacturer's protocol (Sigma-Aldrich; Cat# 23479), as previously described[12]. Nitrate and nitrite content levels of NO metabolites were measured in WT and $ANT2^{-/-}$ primary mouse RPTCs to evaluate endogenous nitrosative stress, and in cultured media to assess exogenous nitrosative stress.

Real-time PCR

mRNA from kidney cortices or primary mouse RPTCs was extracted using a Bio-Tri RNA lysis buffer (Bio-Lab, Israel), followed by DNase I treatment (Thermo Scientific, IL, USA), and reverse transcribed using the qScript cDNA Synthesis kit (Quantabio). Real-time PCR was performed using iTaq Universal SYBR Green Supermix (Bio-Rad, CA) and the CFX connect ST system (Bio-

Rad, CA). The primers used: Monocyte chemotactic protein-1 (*Mcp1)* (5'-GCATTAGCTTCAGATTTA-3', 5'-TTAAAAACCTGCATCGGAACCAA-3'), Lipocalin 2 (*Lcn2)* (5'-AAACAGAAGGCAGCTTTACGA-3', 5'-TCTGATCCAGTAGCGACAGC-3'), Solute Carrier Family 25 Member 4 (*Slc25a4)* (5'- CAGATCCATTGTGTGGTTTAACA-3', 5'-CCTGTTTTCTGTGGGAATCTAAA-3'), Solute carrier family 25 member 31 (*Slc25a31)* (5'-ATGTCGAACGAATCCTCCAAGA-3', 5'- AGCTTCACACGCTCGATGG-3'), Kidney injury molecule-1 *(Kim1)* (5'-TGTCGAGTGGAGATTCCTGGATGGT-3', 5'-GGTCTTCCTGTAGCTGTGGGCC-3'), Cluster of differentiation 36 *(Cd36)* (5'-ATGGGCTGTGATCGGAACTG-3', 5'- GTCTTCCCAATAAGCATGTCTCC-3'), Fatty acid transport protein 2 *(Fatp2)* (5'-ACACACCGCAGAAACCAAATGACC-3', 5'-TGCCTTCAGTGGATGCGTAGAACT-3'), Peroxisome proliferator-activated receptor- γ coactivator-1 α (*Pgc1a*) (5'-AACCACACCCACAGGATCAGA-3', 5'-TCTTCGCTTTATTGCTCCATGA-3'), Dynamin-1-like protein (*Drp1*) (5'-CAGGAATTGTTACGGTTCCCTAA-3', 5'-CCTGAATTAACTTGTCCCGTGA-3'), Mitofusin-1 (*Mfn1*) (5'-CCTACTGCTCCTTCTAACCCA-3', 5'-AGGGACGCCAATCCTGTGA-3'), Mitofusin-2 (*Mfn2*) (5'-AGAACTGGACCCGGTTACCA-3', 5'-CACTTCGCTGATACCCCTGA-3'), and Hypoxia-inducible factor 1-alpha (*Hif1a*) (5'-GGGGAGGACGATGAACATCAA-3', 5'-GGGTGGTTTCTTGTACCCACA-3'). All genes were normalized to Ubiquitin C (*Ubc)* (5'-CCCAGTGTTACCACCAAGA-3', 5'-CCCATCACACCCAAGAACA-3').

Targeted Transcriptomics

The targeted transcriptomics profiling using the nanoString nCounter platform (NanoString Technologies, Inc., Seattle, WA) was done on metabolism-related gene expression. Expression profiling of 768 metabolism-related genes was performed using the nCounter® Metabolic Pathways Panel (NanoString Technologies, Inc., Seattle, WA). Technical and biological normalization against a global geometric mean was performed using nSolver 4.0 software (NanoString Technologies). Briefly, technical normalization was performed using the nSolver software (version 4.0.70) by normalizing the sample target raw counts to the geometric mean of positive control spike-ins. Biological normalization was performed by normalizing the sample target raw counts to the geometric mean of selected housekeeping genes. For pathway analysis, the reference data file, annotated with different functions for 768 genes in the metabolic pathways panel, was obtained using the Advanced Analysis package in the nSolver Analysis Software. The volcano plot was generated based on Student's t-test analysis results for the selected group and plotted using the EnhancedVolcano module in R programming language. The pathway score heatmap and a detailed analysis of the fatty acid oxidation pathway were generated using the seaborn module in Python 3.7. Housekeeping genes used in the assay: *Abcf1, Cog7, Dnajc14, Dhx16, Sdha, Fcf1, Ubb, Oaz1, Nrde2, Mrps5, Tlk2, Agk, Usp39, Polr2a, G6pdx, Tbp, Edc3,* and *Sap130.*

Western Blotting

Kidney or cell homogenates were prepared in a RIPA buffer (25 mM Tris-HCl pH 7.6, 150 mM NaCl, 1% NP-40, 1% sodium deoxycholate, 0.1% SDS). Kidney homogenates were prepared by using the BulletBlender® and zirconium oxide beads (Next Advanced, Inc., NY, USA). Protein

concentrations were measured with the Pierce™ BCA Protein Assay Kit (Thermo Scientific, IL, USA). Samples were resolved by SDS-PAGE (4-15% acrylamide, 150V), and transferred to PVDF or nitrocellulose membranes using the Trans-Blot® Turbo™ Transfer System (Bio-Rad, CA). Membranes were then incubated for 1 h in 5% milk (in 1 × TBS-T) to block unspecific binding. Membranes were incubated overnight in ANT2 (Cell signaling Technology; Cat #14671), ANT4 (Biorbyt; Cat# orb159946), CPT1a (Abcam; Cat# ab128568), Aldolase B (ALDOB) (Abcam; Cat# ab153828), Hexokinase II (HK2) (Cell Signaling Technology; Cat# 2867S), 3-Nitrotyrosine (Abcam; Cat# ab110282), phosphorylated DRP1 (Abcam; Cat# ab193216), total FRP1 (Abcam; Cat# ab5788), PGC1α (Novus biologicals; Cat# NBP1-04676), and MFN2 (Abcam; Cat# ab56889) antibodies at 4 °C. Anti-rabbit/mouse horseradish peroxidase (HRP)-conjugated secondary antibodies were used for 1 h at room temperature, followed by chemiluminescence detection using Clarity™ Western ECL Blotting Substrate (Bio-Rad, CA). Densitometry was quantified using ImageJ software. Quantification was normalized to anti-β actin antibody (Abcam; Cat# ab49900), valosin-containing protein (VCP) (Abcam; Cat# ab204290), voltage-dependent anion channel (VDAC1)/Porin (Abcam; Cat# ab15895), heat shock protein 90 (HSP90) (Abcam; Cat# ab203126) or α-tubulin (Cell Signaling Technology; Cat# 3873S).

Protein Fractionation

Protein fractionation was performed on extracted primary mouse RPTCs using the Cell Fractionation Kit (Abcam; Cat# ab109719). Cytosolic and mitochondrial fractions were verified (**Supplementary Figure 10**), and used for Western blotting. The nuclear fraction of the cells was used for the SIRT1 Activity Assay (Abcam; Cat# ab156065).

Proteomics

Kidney tissues were isolated from RPTC-ANT2$^{-/-}$ mice and their WT littermate controls. Tissues were homogenized on ice in freshly prepared lysis buffer: (8 M urea, 20 mM HEPES, 1 mM sodium orthovanadate, 2.5 mM sodium pyrophosphate, 1mM B-glycerophosphate, 10 µL/mL EDTA-free protease inhibitors cocktail, at pH 8). The homogenates were centrifuged (12,000–13,000 × g for 10 min at 4 °C), the supernatant was collected and the protein concentration was estimated by BCA (Thermo Scientific™; Cat# 23225). Next, the samples were incubated overnight with trypsin at 37 °C and centrifuged (14,000 × g for 45 min). The samples were supplemented with 0.5 M NaCl and centrifuged again (14,000 × g for 25 min). Finally, the samples were acidified with 25% trifluoroacetic acid (TFA) and dried.

The peptides were re-suspended in 2%ACN/H_2O/0.1% Formic acid. Next, 2 µL of the sample was resolved by reverse-phase chromatography on 0.075 × 180-mm fused silica capillaries (J&W) packed with Reprosil reversed phase material (Dr. Maisch GmbH, Germany). The peptides were eluted with a linear 180 min gradient of 5 to 28%, a 15 min gradient of 28 to 95%, and 25 min at 95% acetonitrile with 0.1% formic acid in water at a flow rate of 0.15 µL/min. Mass spectrometry was performed using a Q Executive HFX mass spectrometer (Thermo) in a positive mode using a repetitively full MS scan followed by High Collision Dissociation (HCD) of the 30 most dominant ions selected from the first MS scan.

Mass spectra data were processed using the MaxQuant (Max-Planck-Institute of Biochemistry) computational platform, version 2.0.3.0. Peak lists were searched against Uniprot FASTA sequence database UP000000589 for mice containing 55,341 entries. The search included cysteine carbamidomethylation as a fixed modification, N-terminal acetylation and oxidation of methionine as variable modifications and allowed up to two miscleavages. The 'match-between-

runs' option was used. Peptides with a length of at least seven amino acids were considered and the required fold discovery rate (FDR) was set to 1% at the peptide and protein levels. Relative protein quantification in MaxQuant was performed using the label-free quantification (LFQ) algorithm.

LFQ calls of the two treatments (RPTC-ANT2$^{+/+}$-HFD and RPTC-ANT2$^{-/-}$- HFD) were further analyzed using the Perseus (Max-Planck-Institute of Biochemistry) computational platform, version 1.6.10.43[13]. Proteins were filtered out if they were identified only by site, aligned to the reverse proteins database, or marked as a potential contaminant by the MaxQuant algorithm. LFQ signals were then log2 transformed and only proteins with at least 2 valid values in at least one of the treatment groups were kept, leaving us with 3,018 proteins for further analysis. Missing values were then replaced by randomly selected values from the lower area of the log2 LFQ distribution histogram (AKA Imputation by replacing missing values from the normal distribution, with default parameters). The matrix was exported to a table, which was used for plotting of PCA, heat-map, and volcano plots using in-house R scripts with R version 4.0.4 version and the ggplot2 R graphical package version 3.3.3[14].

A t-test of the comparison between the two groups was carried out to provide 176 significantly differentially expressed proteins (p-value<0.05). Canonical pathway enrichment analysis of the significantly differentially expressed proteins of the RPTC-ANT2$^{+/+}$-HFD vs. RPTC-ANT2$^{-/-}$-HFD comparison (p-value<0.05) was performed using Ingenuity Pathway Analysis (IPA®) (QIAGEN, Inc.). The mass spectrometry proteomics data have been deposited to the ProteomeXchange Consortium via the PRIDE[15] partner repository with the dataset identifier PXD042128.

LC-MS-based metabolomics analysis

Primary mouse WT and ANT2 null RPTCs were isolated and seeded in 6 well plates with complete DMEM for 24 h. Then after, the cells were treated with/without oleate and palmitate (0.1 mM O:P; 2:1, respectively) in DMEM (0.5% FBS; 1% glutamine; 1% P/S; lacking glucose and pyruvate) in the presence of 5 mM U-$^{13}C_6$-labelled D-glucose (Sigma-Aldrich; Cat# 389374) for 3 and 6 h. The plates were washed twice with ice-cold 1 × PBS and supplemented with 500 µL of cold (-20 °C) metabolite extraction solvent (methanol:acetonitrile:water, 5:3:2, respectively), then kept on ice under rotation for 10 min. The cell extracts were collected into microcentrifuge tubes and re-centrifuged at 18,000 × g for 10 min at 4 °C. The supernatants were then transferred to glass HPLC vials and stored at -80 °C prior to LC-MS analysis. Protein concentrations for each well was determined using the Pierce™ BCA Protein Assay Kit (Thermo Scientific, IL, USA).

LC-MS metabolomics analysis was performed as described previously[16]. Briefly, a Dionex Ultimate 3000 high-performance liquid chromatography (UPLC) system coupled to an Orbitrap Q-Exactive Mass Spectrometer (Thermo Fisher Scientific) with a resolution of 70,000 at 200 mass/charge ratio (m/z), electrospray ionization in the HESI source, and polarity switching mode to enable both positive and negative ions across a mass range of 70 to 1000 m/z, was used. The UPLC setup included a ZIC-pHILIC column (SeQuant; 150 mm × 2.1 mm, 5 µm; Merck) with a Sure-Guard filter (SS frit 0.5 µm). Five µL of the cell extracts were injected and the compounds were separated with a mobile phase gradient of 15 min, starting at 20% aqueous (20 mM ammonium carbonate adjusted to pH 9.2 with 0.1% of 25% ammonium hydroxide) and 80% organic (acetonitrile) and terminated with 20% acetonitrile. The flow rate and column temperature were maintained at 0.2 mL/min and 45 °C, respectively, for a total run time of 26 min. All

metabolites were detected using mass accuracy below 5 ppm. Thermo Xcalibur was used for the data acquisition.

Data processing and analysis were performed using TraceFinder 5.2 (Thermo Fisher Scientific) and Metabolite-Auto Plotter 2[17]. The exact mass of the singly charged ion was identified and confirmed by the known retention time using an in-house MS library built by running commercial standards for all detected metabolites. Each identified metabolite intensity was normalized to the protein concentration of each sample. The dynamic changes from 3 to 6 hours of the incorporation of [^{13}C] into labelled metabolites were calculated as follow: M+6 for glucose and fructose-6-phosphate, M+3 for DHAP, PEP, pyruvate, and lactate, M+2 for citrate, *cis*-aconitate, αKG, succinate, malate, glutamate, and aspartate. In addition, the levels of L-carnitine and Deoxycarnitine were measured in RPTCs following 3 h of exposure to O:P treatment.

Statistical analysis

Values are expressed as the mean ± SEM. Unpaired two-tailed Student's *t*-test was used to determine differences between two groups. Results in multiple groups and the time-dependent variables were compared by one-way and two-way ANOVA, respectively, followed by the Brown-Forsythe and Bartlett's test or Tukey's multiple comparisons test, respectively (GraphPad Prism 8). Significance was set at $P<0.05$.

Supplementary References

1. Rubera, I., *et al.* Specific Cre/Lox recombination in the mouse proximal tubule. *J Am Soc Nephrol* **15**, 2050-2056 (2004).
2. Cho, J., *et al.* Mitochondrial ATP transporter Ant2 depletion impairs erythropoiesis and B lymphopoiesis. *Cell Death Differ* **22**, 1437-1450 (2015).
3. Pham, A.H., McCaffery, J.M. & Chan, D.C. Mouse lines with photo-activatable mitochondria to study mitochondrial dynamics. *Genesis* **50**, 833-843 (2012).
4. Kogot-Levin, A., *et al.* Proximal Tubule mTORC1 Is a Central Player in the Pathophysiology of Diabetic Nephropathy and Its Correction by SGLT2 Inhibitors. *Cell Rep* **32**, 107954 (2020).
5. Lee, Y.S., *et al.* Increased adipocyte O2 consumption triggers HIF-1alpha, causing inflammation and insulin resistance in obesity. *Cell* **157**, 1339-1352 (2014).
6. Drori, A., *et al.* Cannabinoid-1 receptor regulates mitochondrial dynamics and function in renal proximal tubular cells. *Diabetes Obes Metab* **21**, 146-159 (2019).
7. Udi, S., *et al.* Proximal Tubular Cannabinoid-1 Receptor Regulates Obesity-Induced CKD. *J Am Soc Nephrol* **28**, 3518-3532 (2017).
8. Knani, I., *et al.* Targeting the endocannabinoid/CB1 receptor system for treating obesity in Prader-Willi syndrome. *Mol Metab* **5**, 1187-1199 (2016).
9. Baraghithy, S., *et al.* Renal Proximal Tubule Cell Cannabinoid-1 Receptor Regulates Bone Remodeling and Mass via a Kidney-to-Bone Axis. *Cells* **10**(2021).
10. Folch, J., Lees, M. & Sloane Stanley, G.H. A simple method for the isolation and purification of total lipides from animal tissues. *The Journal of biological chemistry* **226**, 497-509 (1957).

11. Greenspan, P., Mayer, E.P. & Fowler, S.D. Nile red: a selective fluorescent stain for intracellular lipid droplets. *Journal of Cell Biology* **100**, 965-973 (1985).

12. Tripathi, M.K.*, et al.* The NO Answer for Autism Spectrum Disorder. *Adv Sci (Weinh)* **10**, e2205783 (2023).

13. Tyanova, S.*, et al.* The Perseus computational platform for comprehensive analysis of (prote)omics data. *Nature Methods* **13**, 731-740 (2016).

14. Wickham, H. *ggplot2: Elegant Graphics for Data Analysis*, (Springer-Verlag, New York, 2016).

15. Perez-Riverol, Y.*, et al.* The PRIDE database resources in 2022: a hub for mass spectrometry-based proteomics evidences. *Nucleic Acids Res* **50**, D543-D552 (2022).

16. Mackay, G.M., Zheng, L., van den Broek, N.J. & Gottlieb, E. Analysis of Cell Metabolism Using LC-MS and Isotope Tracers. *Methods Enzymol* **561**, 171-196 (2015).

17. Pietzke, M. & Vazquez, A. Metabolite AutoPlotter - an application to process and visualise metabolite data in the web browser. *Cancer Metab* **8**, 15 (2020).

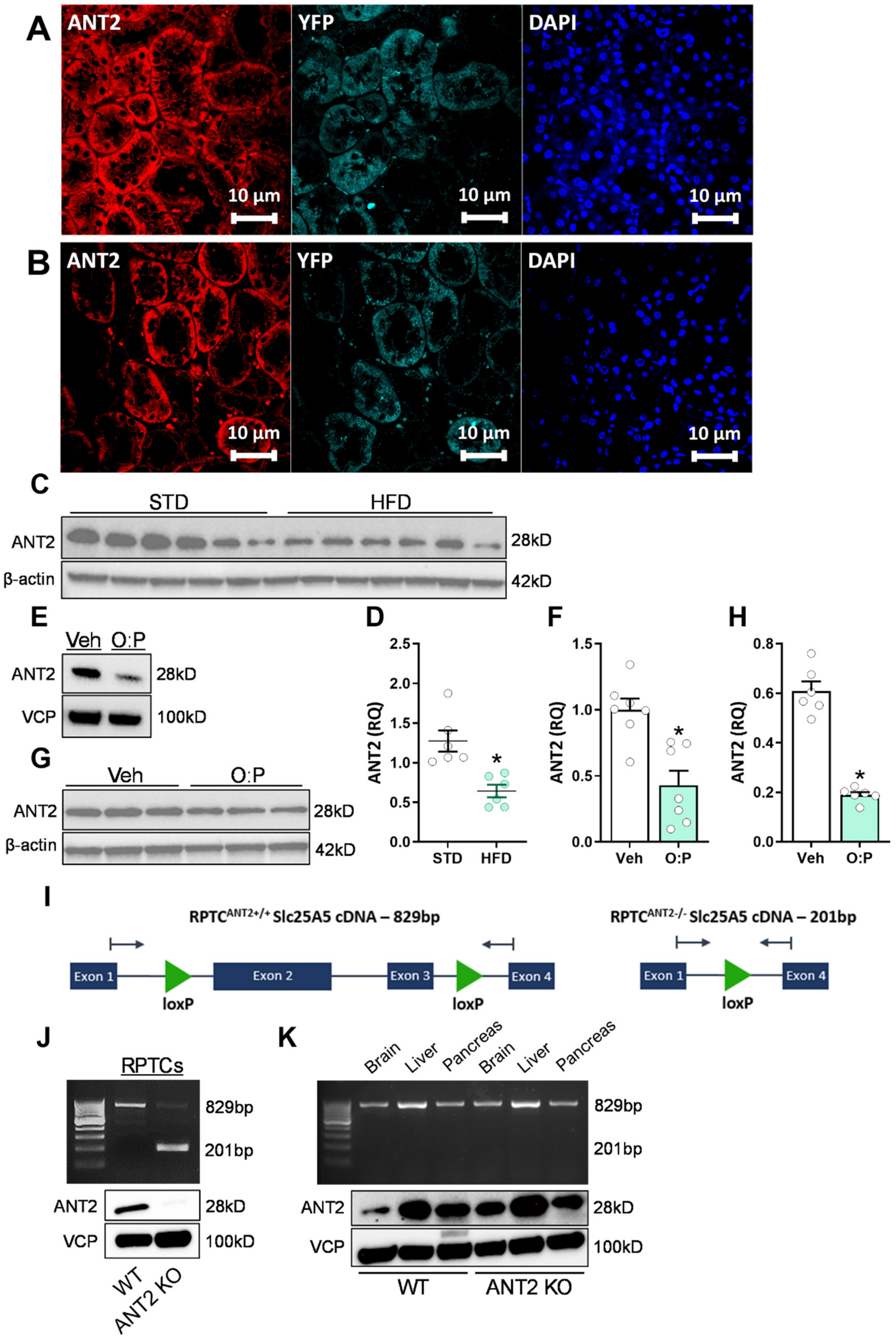
A
ANT2
YFP
DAPI
10 µm
B
ANT2
YFP
DAPI
10 µm
C
STD
HFD
ANT2
28kD
β-actin
42kD
E
Veh
O:P
ANT2
28kD
VCP
100kD
G
Veh
O:P
ANT2
28kD
β-actin
42kD
D
ANT2 (RQ)
STD
HFD
F
ANT2 (RQ)
Veh
O:P
H
ANT2 (RQ)
Veh
O:P
I
RPTC^ANT2+/+ Slc25A5 cDNA – 829bp
Exon 1
Exon 2
Exon 3
Exon 4
loxP
RPTC^ANT2-/- Slc25A5 cDNA – 201bp
J
RPTCs
829bp
201bp
ANT2
28kD
VCP
100kD
WT
ANT2 KO
K
Brain
Liver
Pancreas
829bp
201bp
ANT2
28kD
VCP
100kD
WT
ANT2 KO

Supplementary Figure 1. Generation of RPTC-specific ANT2-null mice.

A, B. Immunofluorescent staining of fixed kidney tissue from Sglt2-Cre;Rosa26-YFP reporter mice fed a standard diet (STD) (**A**) or a high-fat diet (HFD) (**B**) for ANT2 and YFP-marked RPTCs.

C-H. Assessment of ANT2 protein expression levels in kidney tissue lysates from STD- and HFD-fed WT mice (**C, D**), primary mouse RPTCs (**E, F**), and HK-2 cells (**G, H**) treated with a mixture of oleate and palmitate (O:P) in comparison to vehicle-treated controls (n = 6-7 biological replicates in each group). The data represent mean±SEM. *P<0.05 vs. STD or Veh by Student's t-test.

I. Schematic representation of the ANT2 gene with flox sites (green triangles) and forward and reverse primers (arrows). Cre recombination yields a 201 bp cDNA product, while no recombination yields an 829 bp cDNA product.

J-K. Representative PCR (upper panel) and Western blotting (lower panel) analyses conducted on DNA and protein extracted from RPTCs (**J**) as well as brain, liver, and pancreas (**K**), illustrating the disparity in ANT2 expression between WT and ANT2-null mouse strains.

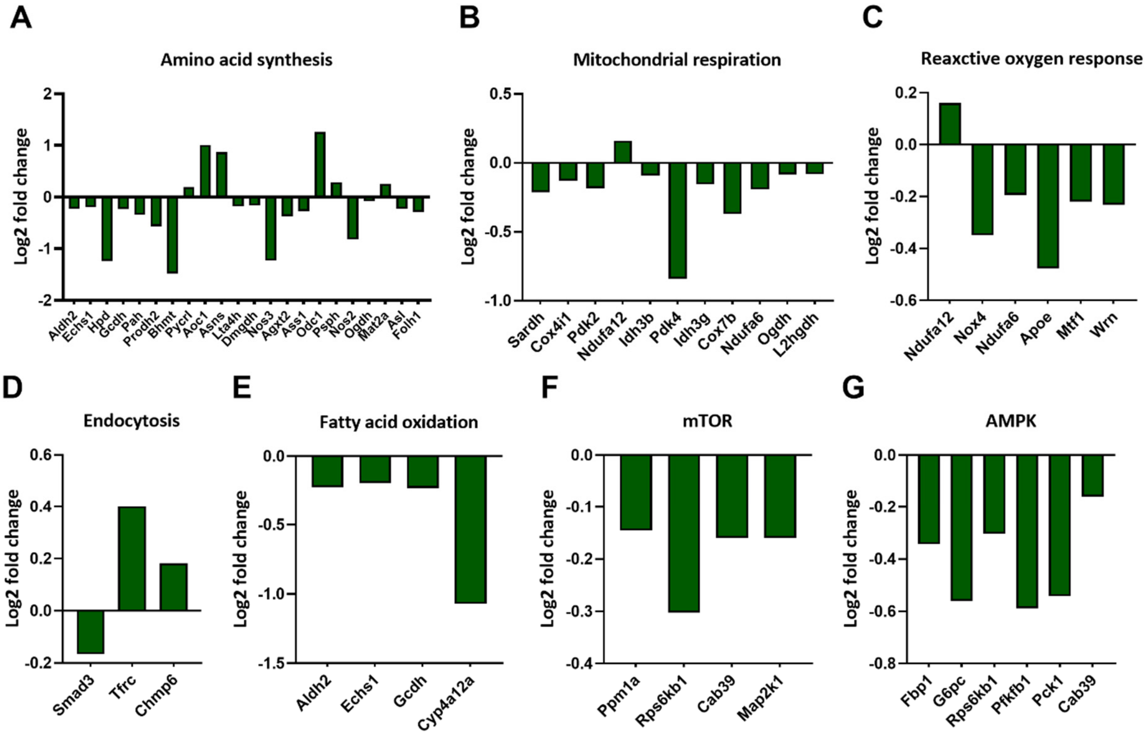

Supplementary Figure 2. Impact of RPTC-ANT2 nullification on transcriptome-level cellular and organelle functions.

Mice lacking RPTC-ANT2 (RPTC-ANT2$^{-/-}$) and their wild-type (WT) littermate controls were subjected to a 24-week high-fat diet (HFD) regimen. Kidney samples from these mice were collected and subjected to targeted large-scale transcriptomics analysis.

A-G. Differential mRNA expression profiles in HFD-fed RPTC-ANT2$^{-/-}$ mice compared to their obese WT littermate controls, categorized by various signaling pathways. These include amino acid synthesis (**A**), mitochondrial respiration (**B**), reactive oxygen response (**C**), endocytosis (**D**), fatty acid oxidation (**E**), mTOR (**F**), and AMPK (**G**) pathways.

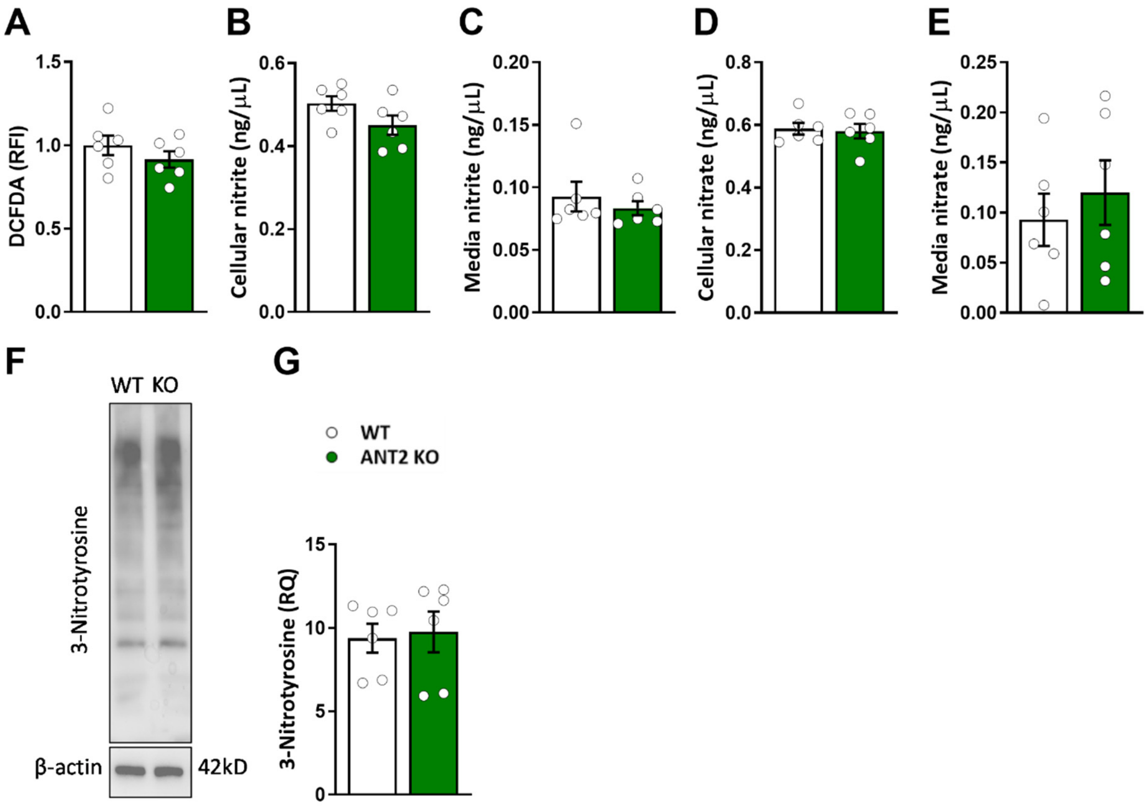

Supplementary Figure 3. Effect of ANT2 nullification on oxidative and nitrosative stress in primary mouse RPTCs.

Primary mouse RPTCs isolated from STD-fed RPTC-ANT2$^{-/-}$ and WT controls were subjected to oxidative and nitrosative stress assessment.

A. Evaluation of reactive oxygen species using DCFDA staining (n = 6 biological replicates in each group).

B-E. Assessment of cellular and media nitrite (**B, C**) and nitrate (**D, E**) levels (n = 6 biological replicates in each group).

F-G. Quantification of 3-Nitrotyrosine levels via Western blotting analysis (**F**) and quantification (**G**) (n = 6 biological replicates in each group).

The data are presented as mean±SEM.

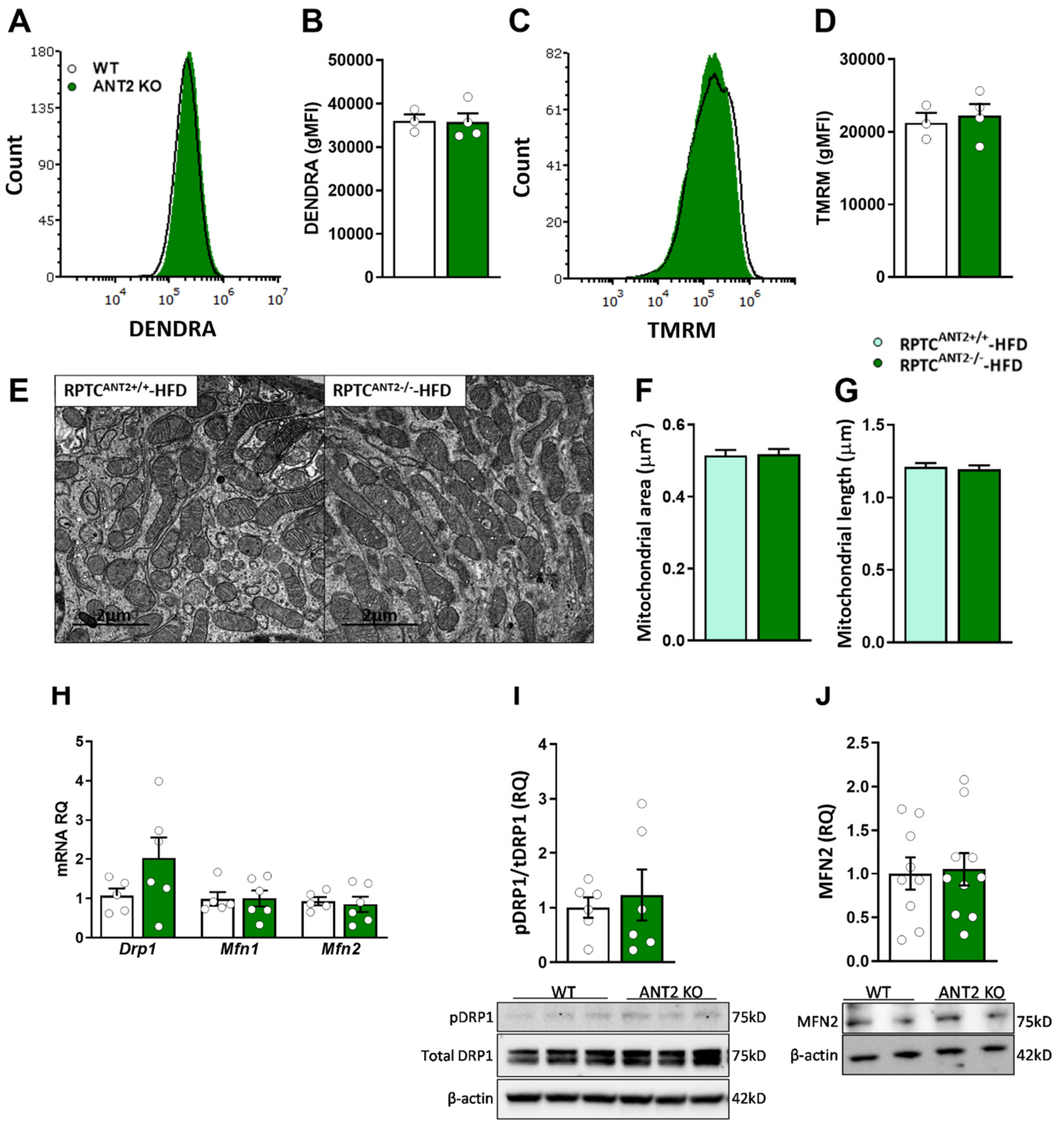

Supplementary Figure 4. Impact of ANT2 nullification in RPTCs on mitochondrial characteristics.

A-D. Assessment of mitogenesis (**A, B**) and TMRM staining for mitochondrial membrane potential (**C, D**) in primary mouse RPTCs isolated from DENDRA-positive ANT2$^{-/-}$ and WT mice fed with STD using FACS (n = 3-4 biological replicates per group).

E-G. Evaluation of mitochondrial area (**F**) and length (**G**) through transmission electron microscopy (**E**) analysis in RPTC-ANT2$^{-/-}$ and WT mice subjected to a high-fat diet (HFD) (n = 571-579 mitochondria per group).

H. Expression of the *Drp1* gene responsible for fission and the *Mfn1/2* genes responsible for fusion in primary mouse RPTCs isolated from STD-fed RPTC-ANT2$^{-/-}$ and WT mice (n = 5-6 biological replicates per group).

I, J. Protein expression of phosphorylated DRP1 (**I**) and MFN2 (**J**) in primary mouse RPTCs isolated from STD-fed RPTC-ANT2$^{-/-}$ and WT mice via Western blotting analysis (n = 6-10 biological replicates per group).

The data are presented as mean±SEM.

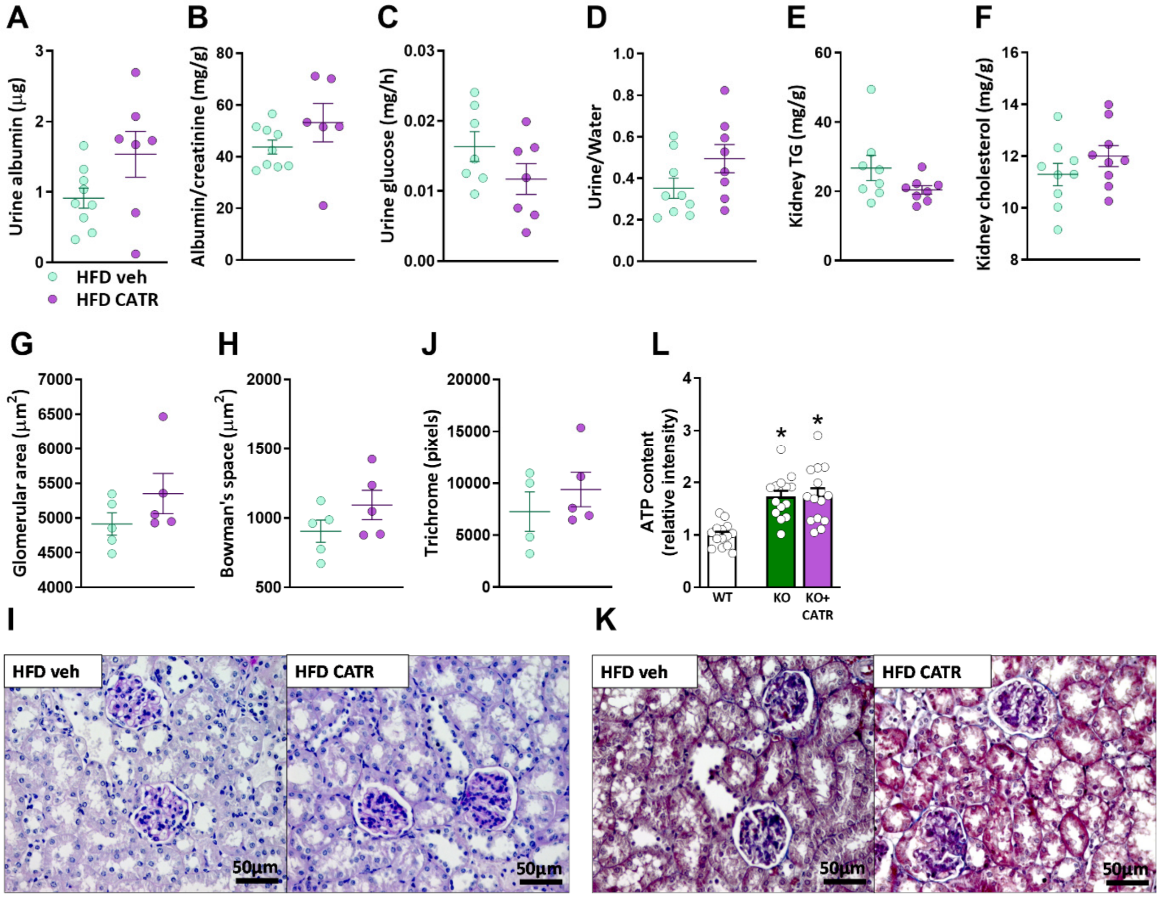

Supplementary Figure 5. Carboxyatractyloside (CATR) treatment does not replicate the positive renal effect of RPTC-ANT2 nullification in high-fat diet (HFD)-fed mice.

C57BL/6J WT mice were fed with a HFD for 28 weeks. During the last 4 weeks of the experiment, the mice received a daily 1 mg/kg i.p. injection of the ANT inhibitor, carboxyatractyloside (CATR) or vehicle.

A-C. Biological measurements of urine albumin (**A**), albumin-to-creatinine ratio (**B**), and urine glucose (**C**) in CATR-treated animals in comparison to vehicle-treated controls (n = 6-9 mice per group).

D. Ratio between urine excreted and water consumed in CATR-treated mice and their vehicle-treated controls (n = 8-9 mice per group).

E, F. Kidney triglyceride (**E**) and cholesterol (**F**) levels in CATR-treated mice and their vehicle-treated controls (n = 8 mice per group).

G-I. Quantification of glomerular (**G**) and Bowman's space (**H**) areas in kidney sections stained with PAS staining (**I**) in CATR-treated mice and their vehicle-treated controls (n = 5 mice per group).

J, K. Quantification of fibrogenesis (**J**) in Trichrome staining kidney slides (**K**) in CATR-treated mice compared to their vehicle-treated controls (n = 4-5 mice per group).

In-vivo data are presented as the mean±SEM.

L. Cellular ATP content in CATR-treated RPTCs lacking ANT2 and their vehicle-treated controls (n = 14-15 biological replicates per group).

In-vitro data are expressed as mean±SEM. *P<0.05 vs. WT assessed using one-way ANOVA.

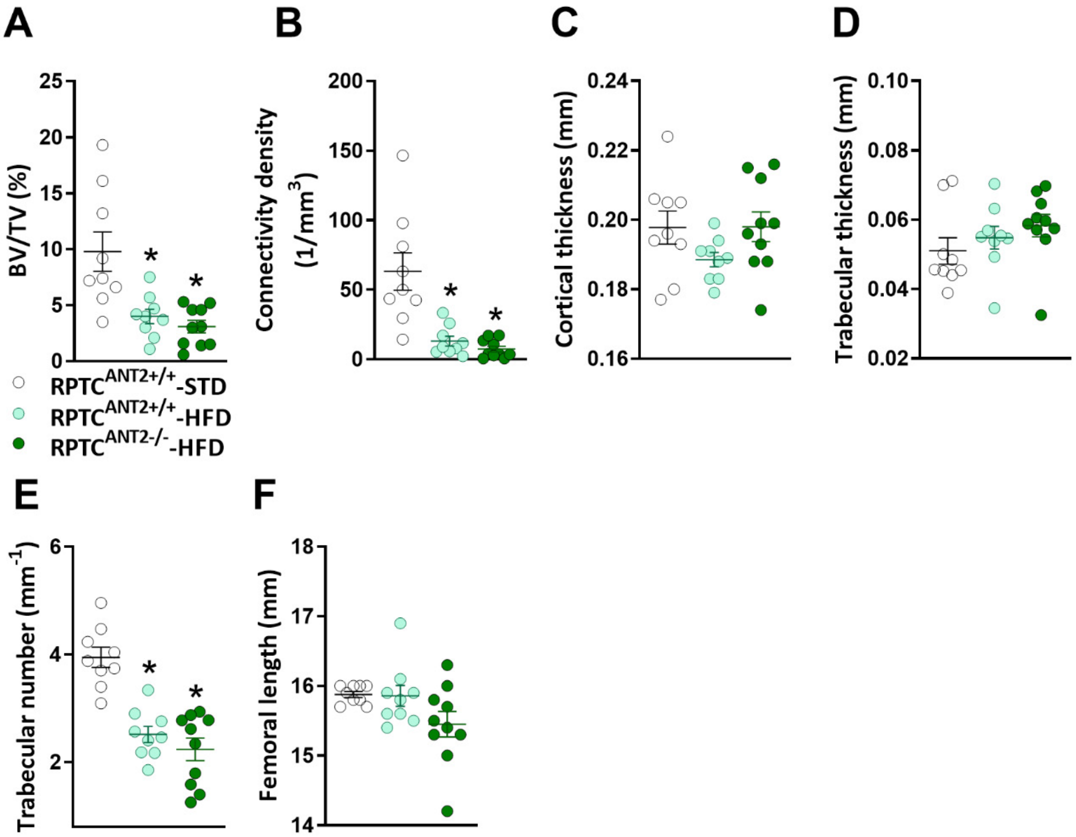

Supplementary Figure 6. RPTC ANT2 deletion does not affect bone morphology.

MicroCT measurements of BV/TV (**A**), connectivity density (**B**), cortical thickness (**C**), trabecular thickness (**D**), trabecular number (**E**), and femoral length (**F**) in RPTC-ANT2$^{-/-}$ mice and their WT controls (n = 9-10 mice per group) fed either a standard diet (STD) or a high-fat diet (HFD) for 24 weeks.

The data represent the mean±SEM. *P<0.05 vs. RPTC$^{ANT2+/+}$-STD by one-way ANOVA.

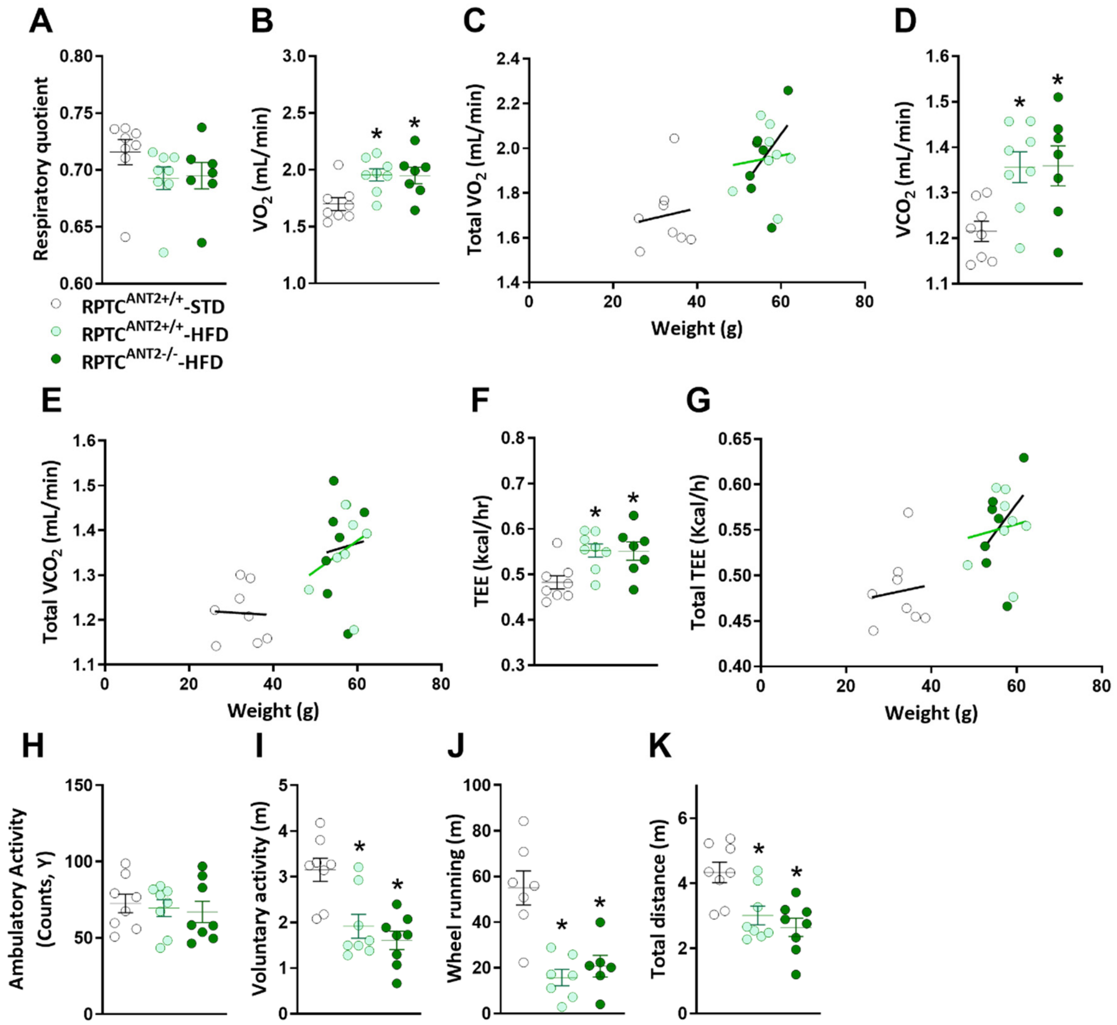

Supplementary Figure 7. RPTC ANT2 nullification does not affect whole-body energy metabolism or activity.

The metabolic and activity profiles of RPTC-ANT2$^{-/-}$ mice and their WT littermate controls fed either a standard diet (STD) or a high-fat diet (HFD) was assessed by the Promethion High-Definition Behavioral Phenotyping System (Sable Instruments). Respiratory quotient (**A**), VO_2 (**B, C**), VCO_2 (**D, E**), total energy expenditure (TEE; **F, G**), ambulatory activity (**H**), voluntary activity (**I**), wheel running (**J**) and total distance (**K**) (n = 7-8 mice per group).

The data represent the mean±SEM. *P<0.05 vs RPTC$^{ANT2+/+}$-STD by one-way ANOVA.

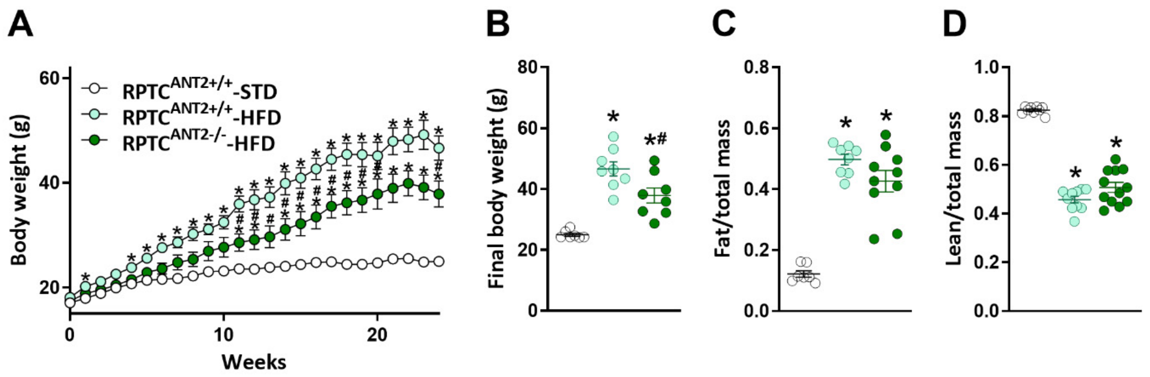

Supplementary Figure 8. Female RPTC-ANT2 KO mice display a similar metabolic phenotype to that of male animals.

A-B. The weekly body weight (**A**) and final (**B**) measurements of female RPTC-ANT2$^{-/-}$ animals and their littermate WT controls fed either a standard diet (STD) or a high-fat diet (HFD) for 24 weeks (n = 7-8 mice per group).

C-D. MRI measurements of relative fat (**C**) and lean (**D**) masses in female animals on STD or HFD (n = 7-10 mice per group).

The data represent the mean±SEM. *P<0.05 vs. RPTC$^{ANT2+/+}$-STD, #P<0.05 vs. RPTC$^{ANT2+/+}$-HFD by one-way ANOVA or two-way ANOVA for time-dependent measurements.

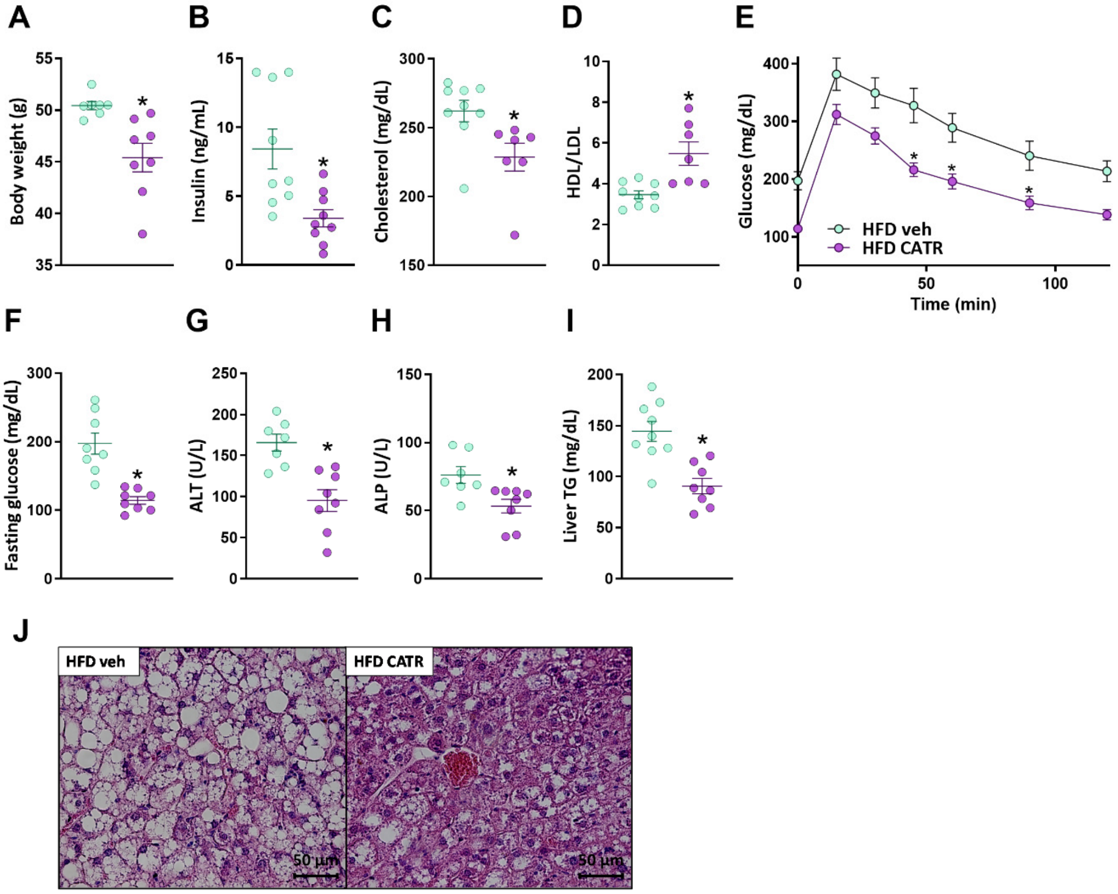

Supplementary Figure 9. Carboxyatractyloside (CATR) treatment improves systemic metabolism in high-fat diet (HFD) fed WT mice similarly to RPTC-ANT2 genetic ablation. C57BL/6J WT mice were fed with a HFD for 28 weeks. During the last 4 weeks of the experiment, the mice received a daily 1 mg/kg i.p. injection of the ANT inhibitor, carboxyatractyloside (CATR) or vehicle. The following parameters were measured at the end of the treatment regimen: (**A**) body weight, (**B**) serum insulin and (**C**) total cholesterol levels, (**D**) the HDL-to-LDL ratio, (**E**) glucose tolerance and (**F**) fasting blood glucose levels, as well as circulating liver enzymes: ALT (**G**) ALP (**H**) and hepatic triglyceride content (TG; **I**). **J.** Representative liver tissue H&E staining of HFD-fed CATR-treated animals and their vehicle-treated controls.

The data represent the mean±SEM from 7-8 animals per group. *P<0.05 vs. HFD veh by Student's t-test or two-way ANOVA for time-dependent measurements.

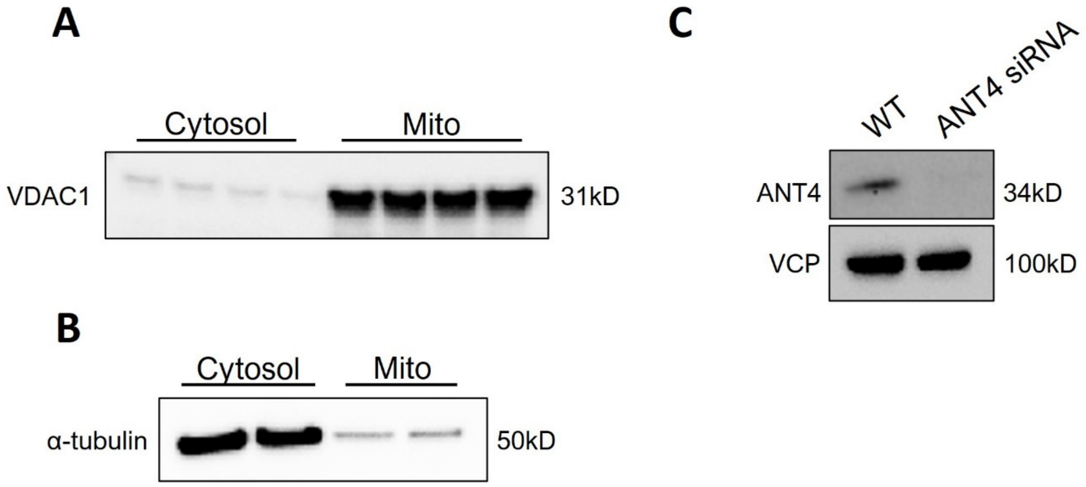

Supplementary Figure 10. Validation of cell fractionation and siRNA transfection.

A. Mitochondrial marker VDAC1 measurement via Western blotting of fractionated primary RPTCs.

B. Cytosolic marker α-tubulin measurement via Western blotting of fractionated primary RPTCs.

C. ANT4 siRNA transfection in primary RPTCs validation by Western blotting.
